# MicroRNA 196a contributes to the aggressiveness of esophageal adenocarcinoma through the MYC/TERT/NFκB axis

**DOI:** 10.1002/1878-0261.70048

**Published:** 2025-09-16

**Authors:** Jesús García‐Castillo, Carlos M. Martínez‐Cáceres, Manuel Bernabé‐García, Vicente Munitiz, David Ruiz de Angulo, Pascual Parrilla, Ángeles Ortiz, Luisa F. Martínez de Haro, María L. Cayuela

**Affiliations:** ^1^ Grupo de Cirugía Digestiva, Endocrina y Transplante de Organos Abdominales Instituto Murciano de Investigación Biosanitaria‐Pascual Parrilla Murcia Spain; ^2^ Grupo de Telomerasa, Cancer y Envejecimiento Instituto Murciano de Investigación Biosanitaria‐Pascual Parrilla Murcia Spain; ^3^ Servicio de Patología Instituto Murciano de Investigación‐Pascual Parrilla Murcia Spain; ^4^ Departamento de Cirugía, Facultad de Medicina Universidad de Murcia Spain; ^5^ Centro de Investigación Biomédica en Red de Enfermedades Raras (CIBERER) Instituto de Salud Carlos III (ISCIII) Madrid Spain

**Keywords:** aggressiveness, Barrett's esophagus, epithelial‐to‐mesenchymal transition, esophageal adenocarcinoma, miR‐196a

## Abstract

Barrett's esophagus (BE) is a premalignant lesion that can lead to an invasive esophageal adenocarcinoma (EAC), a type of cancer that usually has a poor outcome. We have previously described a set of four microRNAs (miR‐192, 194, 196a and b) that are markers of the disease's progression. To determine whether these miRNAs might also be drivers of invasive EAC development, their overexpression in EAC cells was analyzed. Only the overexpression of miR‐196a and miR‐196b induced a phenotype switch in non‐invasive EAC cells, resembling epithelial‐to‐mesenchymal transition (EMT). The overexpression of miR‐196a promoted EMT and increased cell motility and NFκB signaling. Mechanistically, miR‐196a targets the inhibitor of NFκB alpha *NFKBIa* and also leads to c‐MYC protein accumulation by down‐regulating *VCP* expression. This in turn up‐regulated *TERT* expression and reinforced NFκB signaling. NFκB signaling, TERT, and c‐MYC inhibition resulted in a reversed‐EMT phenotype, with decreased EMT hallmarks and cell motility in miR‐196a overexpressing cells. Finally, an immunohistochemical analysis of BE tissue samples showed that c‐MYC, TERT, and NFκB signaling increased in BE patients who developed EAC, more so than in patients that did not. The high expression of miR‐196a induces aggressive features in non‐invasive EAC cells. These effects are dependent on the c‐MYC/TERT/NFκB signaling molecular axis. BE patients and non‐invasive EAC patients with high miR‐196a expression could benefit from therapeutic interventions to prevent EMT or activation of the molecular pathway described in this study.

## Graphical abstract

mir‐196a promotes Esophagus Adenocarcinoma aggressiveness. On one hand, mir‐196a targets the valosin‐containing protein (VCP) mRNA, causing the accumulation of c‐MYC protein that leads to high amounts of TERT. On the other hand, mir‐196a targets the inhibitor of NFκB (NFKBIA). This, along with TERT, increases NFKB signaling and SNAIL1 transcription, provoking an epithelial‐to‐mesenchymal transition (EMT) and tumor aggressiveness.

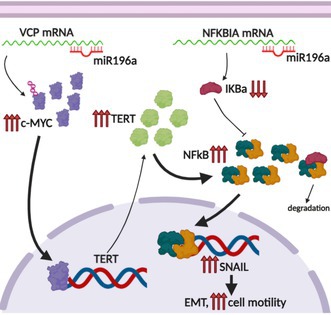

**Abbreviations**
ANOVA: analysis of variance
ATCC: American Type Culture Collection
BE: Barrett's esophagus
*CDH1*
: *e‐cadherin*
DMEM: Dulbecco's Modified Eagle Medium
DMSO: dimethyl sulphoxide
EAC: esophageal adenocarcinoma
ECACC: European Collection of Authenticated Cell Cultures
EMT: epithelial‐to‐mesenchymal transition
FBS: Fetal bovine serum
GAPDH: glyceraldehyde 3‐phosphate dehydrogenase
HGD: high‐grade dysplasia
IKBα: inhibitor of NFκB
miRNAs: microRNAs
PBS: phosphate‐buffered saline
QTRAP: quantitative telomere repeats amplification protocol
RT‐qPCR: real‐time quantitative PCR
TCGA: The Cancer Genome Atlas
TERC: telomerase RNA component
TERT: telomerase reverse transcriptase
TERT‐DN: TERT dominant negative
UBE2C: ubiquitin‐conjugating enzyme E2 C
UBE2Z: ubiquitin‐conjugating enzyme E2 Z
UICC: Union for International Cancer Control
UTR: untranslated region
VCP: valosin‐containing protein
*VIM*
: *VIMENTIN*

## 1 Introduction

Esophageal cancer may have an epithelial, neuroendocrine, lymphoid, or mesenchymal origin. The majority of esophageal tumors are derived from epithelial cells, with two main histological types: squamous cell carcinoma and adenocarcinoma (EAC). EAC is the predominant type in western countries, and its prevalence is increasing [1]. Despite success in cancer diagnosis and treatment, EAC remains a poorly treatable type, with significant mortality after surgery, the most used therapy.

Barrett's esophagus (BE), which is the precursor lesion for EAC, consists of a metaplastic transformation in which the normal esophageal squamous epithelium is replaced by columnar epithelia with goblet cells. Barrett's metaplasia arises from a group of p63^+^KRT5^+^KRT7^+^ transitional basal cells at the esophagus‐stomach squamous‐columnar junction [2]. BE patients have a 30–40 fold increased risk of developing EAC [3]. Gastro‐esophageal reflux disease is the only known risk factor for BE, with a relevant role of biliary‐pancreatic‐duodenal reflux in its pathogenesis [4]. BE progresses to EAC through a well‐known sequence of BE metaplasia/low‐grade dysplasia/high‐grade dysplasia/EAC. Currently, endoscopic with biopsy and regular lesion monitoring is the standard for BE management. However, given that repeated endoscopic interventions are considered an invasive procedure and that only 0.55% of BE patients develop EAC, the cost‐effectiveness of endoscopic surveillance and biopsy has been questioned [5, 6]. Hence, it is necessary to stratify BE patients with a high risk of developing EAC. MicroRNAs (miRNAs) are small non‐coding RNAs of 18–25 nucleotides, which regulate gene expression by binding to the 3′ untranslated region of target mRNA in order to inhibit translation and mediate their degradation [7]. These miRNAs regulate multiple cellular processes such as growth, differentiation, and migration and are very frequently deregulated in cancer. miRNAs are increasingly used in clinical practice as cancer biomarkers since they are stable in formalin‐fixed biopsies and in blood. Furthermore, they can be obtained by techniques of low invasiveness [8]. This means that miRNA biomarkers would be particularly valuable in BE and EAC, a type of cancer that is associated with long monitoring periods and costly and/or invasive methodologies. To date, a number of miRNAs have been reported to be differentially expressed in BE and during its progression to EAC, and they have been proposed as potential biomarkers for the diagnosis and surveillance of BE (reviewed comprehensively by Clark et al. [9]). The vast majority of these studies are based on comparisons of miRNA expression in biopsies from BE or EAC patients, accompanied by a limited number of validation experiments, while conclusions have been drawn by speculating about the function of the predicted miRNA targets in the disease. For instance, the miR200 family is known to inhibit epithelial‐to‐mesenchymal transition (EMT) in breast cancer cells [10] and has been shown to be down‐regulated in the neoplastic transformation of BE to EAC [11], but no study has identified a target or provided insights into the molecular mechanisms or function of these miRNAs in the ethiopathology of BE/EAC.

We have previously identified a group of four miRNAs (miR‐192, 194, 196a, and 196b) that were overexpressed in biopsies of BE patients who developed EAC, but not in BE patients who did not. Moreover, it was demonstrated that these 4 miRNAs are good markers of disease progression [12]. Therefore, in the present study, we wished to ascertain whether the miRNAs are also causally involved in the progression of the disease and, if so, to characterize the function and molecular mechanisms of action of these miRNAs in BE/EAC. We found that the overexpression of miR‐196 family members resulted in an induction of the EMT process and increased cell motility in high‐grade dysplasia/EAC *in situ* cells, as well as in cells of adenocarcinoma of the esophagus/cardia junction, features of tumor cells with high metastatic potential. It was found that these processes are mediated by a MYC/TERT/NFκB molecular axis, whose inhibition results in a decrease in the aggressiveness of EAC cells. Finally, and very importantly, we show that this molecular pathway is operating in patients of BE esophagus time before tumorigenesis occurs.

## 2 Materials and methods

### 2.1 Cell culture

The immortalized, non‐transformed esophagus epithelium cell line Het‐1A (RRID CVCL_3702) was obtained from the American Type Culture Collection (ATCC, cat. CRL‐2692). The high‐grade dysplasia/esophageal adenocarcinoma *in situ* OE33 cell line (RRID CVCL_0471), and the adenocarcinoma of the esophageal/cardia junction OE19 cell line (RRID CVCL_1622) were both obtained from the European Collection of Authenticated Cell Cultures (ECACC, cat. 96070808 and cat. 96071721, respectively). Cells were grown in Dulbecco's Modified Eagle Medium (DMEM, Biowest, Nuaillé, France) supplemented with 10% Fetal Bovine Serum (FBS, Lonza), 1% Penicillin/Streptomycin mix (Lonza, Basel, Switzerland) at 37 °C in a humidified incubator with 5% CO_2_.

Mycoplasma‐free conditions were tested every 3 months using the MycoProbe Mycoplasma Detection kit (R&D Systems, Minneapolis, MN, USA), and all experiments were performed with mycoplasma‐free cells. Cell lines have been authenticated within the last 3 years in the Cell and Tissue Culture facility of the University of Murcia (Molecular Biology Service, Murcia, Spain) using the AmpFLSTR™ Identifiler™ Plus PCR Amplification Kit.

When indicated, OE33 cells were treated for 18 h (h) with the NFκB signaling inhibitor NAI (10 μm, Calbiochem cat. 481406, San Diego, CA, USA), TERT inhibitor BIBR1532 (20 μm, Santa Cruz Biotechnology cat. sc‐203843, Dallas, TX, USA) and MYC inhibitor 10074‐G5 (50 μm, ApexBio cat. C5722, Houston, TX, USA) or with dimethyl sulfoxide (DMSO) as a control.

### 2.2 Transient transfection

Cells were transiently transfected with 100 pmol of miR‐192, 194, 196a, and 196b Pre‐miR miRNA precursors (Ambion, Austin, TX, USA) using Lipofectamine 2000 Reagent (ThermoFisher Scientific, Waltham, MA, USA) following the manufacturer's instructions.

Cells were transiently transfected with 100 pmol of control siRNA, TERC siRNA, or TERT siRNA (all from Santa Cruz Biotechnology) using Lipofectamine RNAiMAX Reagent (ThermoFisher Scientific) following the manufacturer's instructions.

### 2.3 Stable gene expression

A DNA fragment containing the miR‐196a gene sequence was amplified from genomic DNA using the primers shown in Table S1 and standard PCR techniques. The fragment was then cloned in the ApaI site of the pRetroX‐Tre3G vector (Clontech, San Jose, CA, USA). The construct or the vector without insert (as control) was transfected into OE33 and OE19 cells using Lipofectamine 2000 Reagent. 48 h later, cells were selected for stable expression by adding 1 μg·mL^−1^ puromycin into the medium.

pBABE‐puro, pBABE‐puro‐hTERT, and pBABE‐puro‐DN‐hTERT were transfected into OE33 cells using Lipofectamine 2000 Reagent. 48 h later, cells were selected by adding 1 μg·mL^−1^ puromycin into the medium. pBABE‐puro was a gift from Hartmut Land & Jay Morgenstern, and Bob Weinberg (Addgene plasmid #1764, Watertown, MA, USA). pBABE‐puro‐hTERT and pBABE‐puro‐DN‐hTERT were both gifts from Bob Weinberg (Addgene plasmids #1771 and 1775).

### 2.4 Cell proliferation assay

Cell proliferation assays were performed using the Cell Proliferation Reagent WST‐1 (Roche, Basel, Switzerland) following the manufacturer's instructions. Briefly, cells were seeded in 96 well plates, and at the indicated times, WST‐1 reagent was added to the well, and cells were further incubated at 37 °C for 4 h. Absorbance at 450 and 620 nm was measured, and the proliferation rate at each time point was determined as follows: (Abs450‐Abs620)/(Abs450‐Abs620)To.

### 2.5 Analysis of gene expression

Cells were harvested, washed twice in phosphate‐buffered saline (PBS) and total RNA was extracted from cells using the RNeasy Mini kit (Qiagen, Venio, The Netherlands) or Direct‐zol RNA Miniprep kit (Zymo Research, Irvine, CA, USA) following the manufacturer's instructions. cDNA was synthesized using 0.1–2 μg of RNA using the Superscript IV VILO MasterMix (ThermoFisher Scientific). Real‐time quantitative PCR (RT‐qPCR) was performed with a StepOnePlus instrument (Applied Biosystems, Waltham, MA, USA) using SYBR® Premix Ex Taq (Perfect Real Time) (Takara, San Jose, CA, USA). Reaction mixtures were incubated for 30 s (s) at 95 °C, followed by 40 cycles each of 5 s at 95 °C and 20 s at 60 °C, followed by a melting curve. Glyceraldehyde 3‐phosphate dehydrogenase (*GAPDH*) content in each sample was used for normalization of gene expression. The primers used are shown in Table S1. For miRNA expression analysis, cDNA was synthesized from 0.5 to 2 μg of RNA using the miScript II RT kit (Qiagen). RT‐qPCR was carried out using miScript SYBR Green PCR kit (Qiagen) along with miScript Primer Assays for miR192, miR194, miR196a, miR196b, and U6 snRNA (all from Qiagen). Reaction mixtures were incubated for15 minutes at 95 °C, followed by 40 cycles each of 15 s at 94 °C, 30 s at 55 °C, and 30 s at 70 °C, and followed by a melting curve. U6 expression was used for normalization. In all cases, relative expression was determined using the comparative *Ct* method (2^−ΔCt^) and each PCR was performed in triplicate.

### 2.6 Western blot

Cells were harvested, washed twice in PBS, and lysed in RIPA buffer (50 mm Tris‐HCl pH 7.4, 150 mm NaCl, 1 mm EDTA, 0.5 mm DTT, 0.1% SDS, 1% NP‐40, 0.5% sodium deoxycholate) containing a protease inhibitor cocktail (Sigma, Burlington, MA, USA). The samples were then incubated for 30 min on ice with frequent vortexing and centrifuged for 20 min at 4 °C, followed by determining the protein concentration of the supernatant. The proteins were subjected to polyacrylamide gel electrophoresis, transferred to nitrocellulose membranes (BioRad, Hercules, CA, USA) and blotted using 1 : 1000 anti E‐CADHERIN mouse monoclonal antibody (GT477, GeneTex, Irvine, CA, USA), 1 : 3000 anti SNAIL1 rabbit polyclonal antibody (Novus Biologicals NBP2‐29626, Centennial, CO, USA), 1 : 1000 anti VIMENTIN mouse monoclonal antibody (GT812, GeneTex or PA5‐27231, Invitrogen, Waltham, MA, USA), 1 : 500 anti c‐MYC mouse monoclonal antibody (9E10, Santa Cruz Biotechnology), 1 : 500 of anti TERT mouse monoclonal antibody (600‐401‐252, Rockland Immunochemicals, Limerick, PA, USA), anti‐phospho‐NFkB p65 (Ser276) rabbit antibody (Santa Cruz Biotechnology, sc‐101749) and 1 : 5000 anti β‐actin HRP‐conjugated mouse monoclonal antibody (C4, Santa Cruz Biotechnology).

### 2.7 Scratch‐wound assay

Confluent cell monolayers were scratch‐wounded with a blue Gilson pipette tip and further cultured for 18 h in the presence of the indicated inhibitors or DMSO. Pictures were captured by a Nikon Eclipse Ti microscope, equipped with a Nikon DS‐R12 camera. Migration distances at T0 and 18 h post wounding were measured using nis‐element br software (Nikon, Tokyo, Japan). The percentage of wound healed was calculated as follows:

$$\begin{array}{l} \left(\text{wound width}\;\mathrm{at}\;T0-\text{wound width}\;\mathrm{at}\;18\;h\right)/\text{wound width}\\ \;\mathrm{at}\;T0\times 100. \end{array}$$

### 2.8 Transwell chamber assays

Cells (5 × 10^4^) were plated onto serum‐coated transwells (8‐μm pore membranes, Nunc). After 72 h, the cells on the upper side of the membrane were removed with a cotton swab, and the migrated cells on the lower side were fixed with 2.5% glutaraldehyde for 20 min at room temperature. Finally, the membranes were excised and mounted on glass slides. Cells on the lower side of the membranes were counted in a Nikon Eclipse Ti microscope equipped with a Nikon DS‐R12 camera, assessing five random fields at ×20 magnification.

### 2.9 Luciferase assay

For luciferase reporter assays, cells were transfected with 4xNFκB Luc plasmid (firefly luciferase, a gift from Johannes A. Schmid, Addgene plasmid #111216), along with pRL‐CMV (Renilla luciferase, Promega, Madison, WI, USA) and the indicated constructs using Lipofectamine 2000.

For VCP reporter assay, a sequence containing the 3′ untranslated region (UTR) of VCP mRNA was amplified with primers harboring XbaI restriction sites using standard PCR techniques. This fragment was then digested with XbaI and cloned into the XbaI site of pRL‐CMV, downstream of *Renilla* luciferase. For deleting miR‐196a putative binding sequences in the 3′ UTR of VCP mRNA, binding sequences deleted primers were designed and a 2‐step overlapping PCR strategy was used. All primers are shown in Table S1.

At the indicated times after transfection, cells were washed in PBS, harvested, and assayed for luciferase activity using the Dual‐Glo Luciferase Assay System (Promega) following the manufacturer's instructions.

### 2.10 Immunohistochemistry

All the samples were obtained following the Biobanco guides of the Red de la Región de Murcia (BIOBANC‐MUR, member of the Spanish Biobank Network, ISCIII) concerning patient biopsies and storage. 4‐μm sections from formalin‐fixed and paraffin‐embedded samples were taken from all the patients. After deparaffination and rehydration, a heat‐induced demasking antigen procedure (High pH demasking antigen solution, Dako, Glostrup, Denmark) was followed. Endogenous peroxidase was then blocked, followed by 1 h incubation with primary antibodies at 37 °C (1 : 100 anti c‐MYC rabbit polyclonal antibody Abcam Ab32072, Cambridge, UK; 1 : 500 anti TERT rabbit polyclonal antibody Abcam Ab21665; 1 : 200 anti‐phospho‐NFκB p65 Ser276 rabbit polyclonal, Santa Cruz Biotechnology sc‐101749). Sections were then incubated with secondary labeled polymer (Vector Labs, Newark, CA, USA) for 20 min, and the immunoreaction was revealed with 3–3′ diaminobencidine. The sections were hematoxylin counterstained, dehydrated, cleared, and mounted in permanent medium. Samples were analyzed in a Zeiss Axio Scope A1 optic microscope equipped with an Axiocam 506 camera (Carl Zeiss, Oberkochen, Germany). Images were processed using zeiss zen 3.0 software (Carl Zeiss), and positive cells were counted by assessing five random fields at 40× magnification.

### 2.11 Statistical analysis

All the data presented are the average of at least two independent experiments. Quantitative data are represented as mean + standard error of the mean. Data were analyzed by analysis of variance (ANOVA) and a Bonferroni post‐test. The difference between two samples was analyzed by means of the Mann–Whitney test, and differences in the proliferation curves were analyzed by a Kruskal–Wallis test (plus Dunn's post‐test). Correlation goodness was obtained by computing two‐tailed non‐parametric Spearman correlation coefficients. In all the figures, *P < 0.05, **P < 0.01 and ***P < 0.001. All the statistical analyses were performed using graphpad prism software (Boston, MA, USA).

### 2.12 Ethics declaration

All experiments using human samples were performed adhering to the ‘Declaration of Helsinki’. Patient material was collected at the University Hospital Virgen de la Arrixaca in Murcia before 2007. A written informed consent was obtained from all the participants in the study. The use of the human samples for research purposes was authorized by the Bioethics Committee of University Hospital Virgen de la Arrixaca (2009‐1‐4‐HCUVA).

## 3 Results

### 3.1 miR‐196a induces EMT and increases cell motility in EAC cells whereas it did not in non‐transformed esophagus epithelial cells

We have previously reported on a group of four miRNAs (miR‐192, 194, 196a and b), the high expression level of which in patient biopsies of Barrett's esophagus (BE) metaplasia correlated with the subsequent development of esophageal adenocarcinoma (EAC), making them markers of disease progression [12]. To determine whether these miRNAs might also be a cause of disease progression, they were overexpressed (individually or all together) in the OE33 cell line, which is a high‐grade dysplasia (HGD)/EAC *in situ* cell line established from a non‐invasive stage IIA (UICC) EAC that had developed from a Barrett's metaplasia [13]. Overexpression of both miR‐192 and 194 (individually or together) killed the cells, whereas the overexpression of miR‐196a and miR‐196b had no effect on cell proliferation compared with the control (Fig. S1A–E). Importantly, while control cells grew in closely joined cell islets with tight junctions among them and epithelial in shape (typical growth of a non‐aggressive epithelial tumor cell line), cells overexpressing miR‐196a and miR‐196b showed a phenotype of a mesenchymal shape, with cellular protrusions and loose junctions (Fig. S1F–H).

**Fig. 1:**
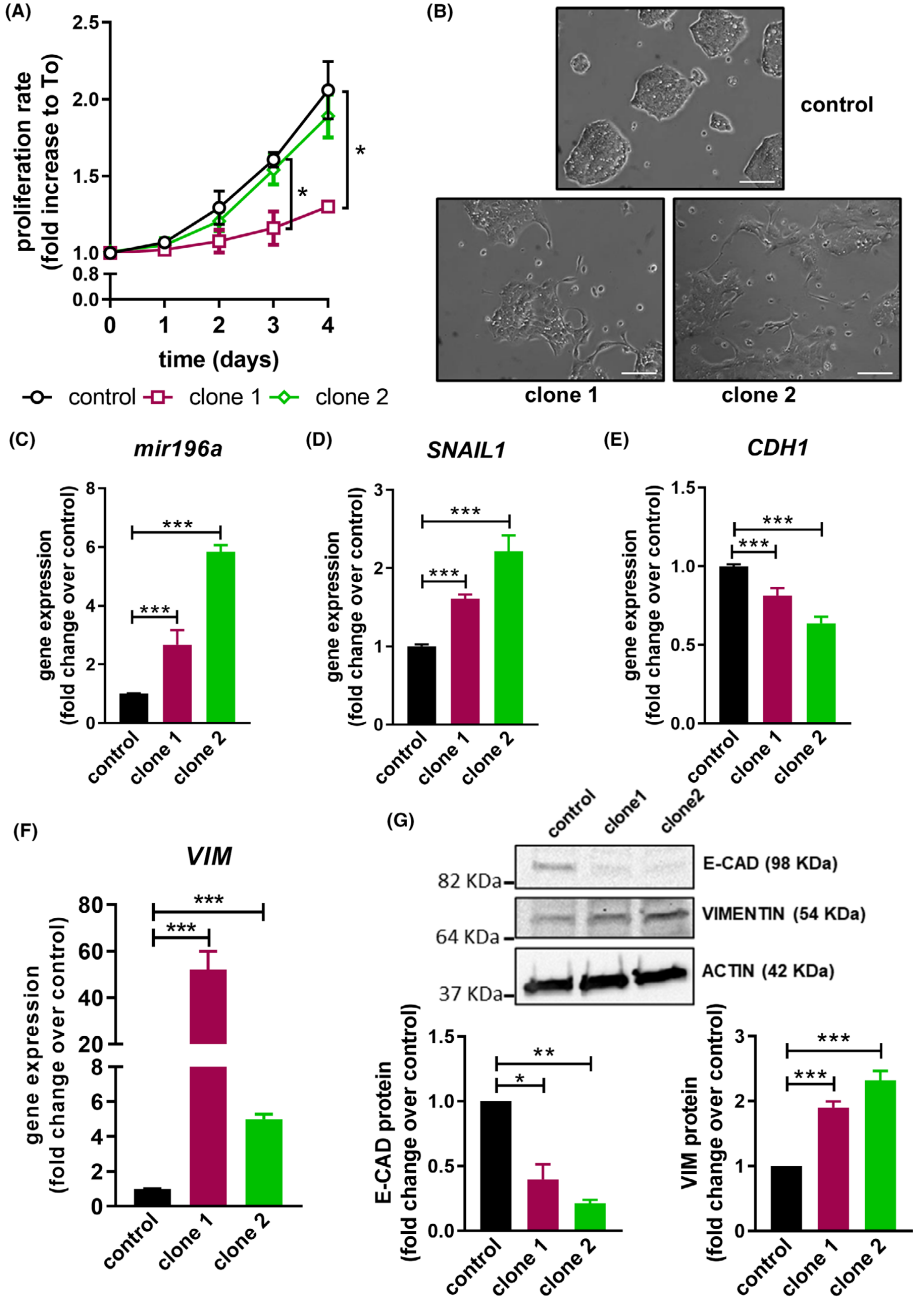
microRNA‐196a (miR‐196a) induces epithelial‐to‐mesenchymal transition (EMT) in Esophageal Adenocarcinoma cells. (A) Proliferation rates of control OE33 cells and two clones stably expressing miR‐196a. Cells were seeded in 96 well plates. At the indicated time points, WST‐1 reagent was added to the medium and after 4 h absorbance at 450 nm was measured. (B) Images of the phenotype switch of OE33 miR‐196a overexpressing cells. Scale bar, 100 μm. (C–F) Gene expression by qPCR of miR‐196a, and the EMT markers *SNAIL1*, *CDH1* and *VIMENTIN* (*VIM*) in control cells and OE33 miR‐196a overexpressing clones. (G) Western blots showing protein expression of the EMT markers E‐CADHERIN and VIMENTIN in control and OE33 miR‐196a overexpressing clones. Data are mean + standard error of the mean of three independent experiments. *P < 0.05, **P < 0.01 and ***P < 0.001 for Kruskal–Wallis test, plus Dunn's post‐test in A and for analysis of variance (ANOVA), plus Bonferroni post‐test in (C–G).

To further confirm this observation, we obtained stable OE33 cells overexpressing miR‐196a. These cells showed no increase in proliferation rates (even clone 1 proliferated at lower ratios) compared with the control, whereas the phenotype switch was quite evident in both clones (Fig. 1A–C). This phenotype, protrusions and loose junctions, is typical of epithelial‐to‐mesenchymal transition (EMT), a process that develops in epithelial tumors that acquire aggressive traits [14]. Consistently, the overexpression of miR‐196a up‐regulated the amount of the EMT‐driver transcription factor *SNAIL1*, down‐regulated the expression of the epithelial marker *CDH1*, and increased the mesenchymal marker *VIMENTIN* at both messenger RNA (mRNA) and protein levels (Fig. 1D–G). We also stably overexpressed miR‐196a in the OE19 cell line, which is a stage III (UICC) adenocarcinoma of the esophageal/cardia junction cells, with moderate differentiation [13] (Fig. S1I). Consistently, proliferation did not change in the OE19 cells overexpressing mir196a (Fig. S1J), and the expression of *SNAIL1* and *VIMENTIN* genes was up‐regulated, and *CDH1* mRNA levels were reduced (Fig. S1K–M).

**Fig. 2:**
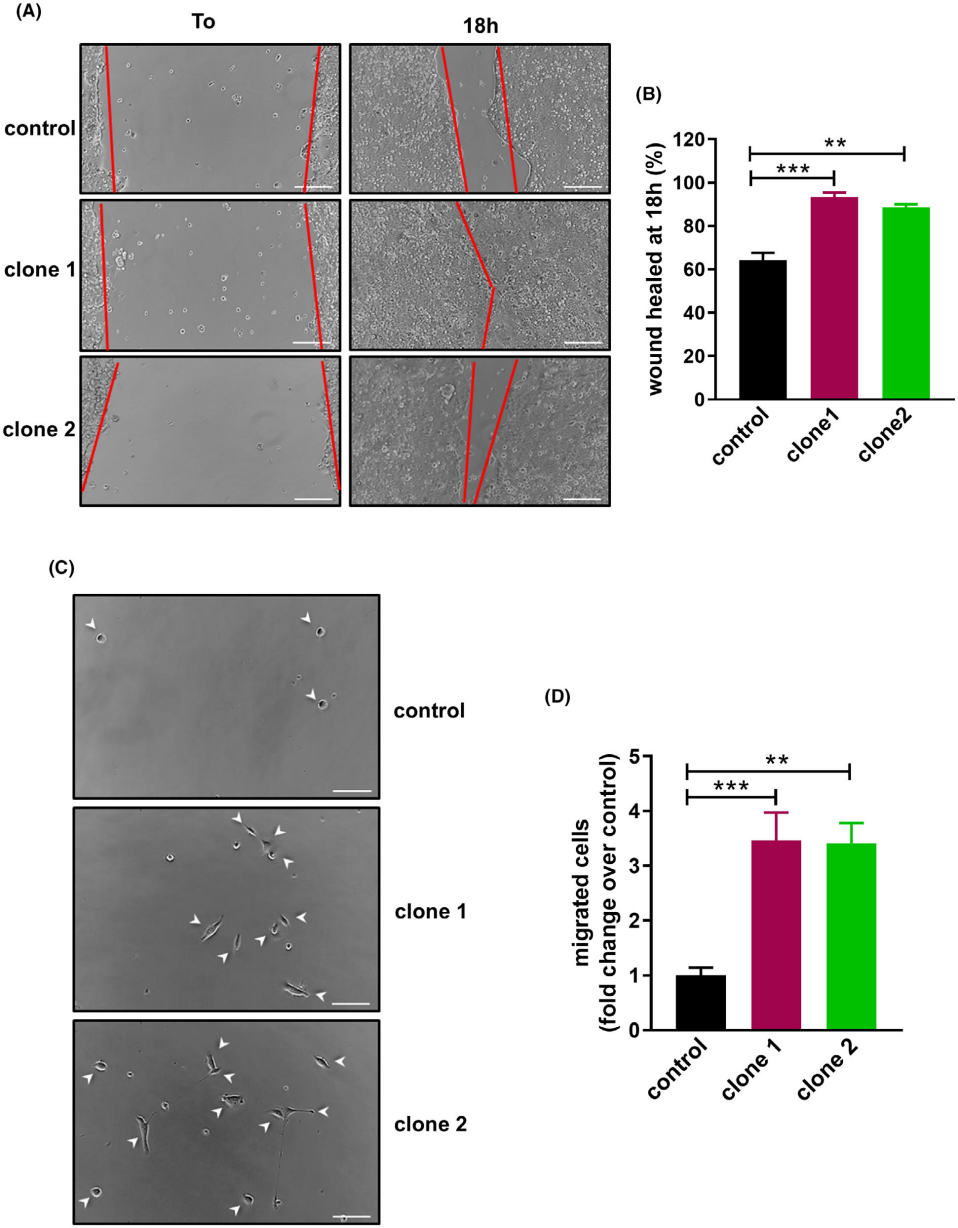
microRNA‐196a (miR‐196a) increases cell motility of Esophageal Adenocarcinoma cells. (A, B) Images of scratch‐wound assay of OE33 control and miR‐196a overexpressing clones and their quantification. Cells were seeded in 6‐well plates, grown to confluence and scratched using a micropipette tip (T0). After 18 h of growth, wound pictures were acquired. Red lines indicate the border of the cell layer. Scale bar, 200 μm. (C, D) Pictures of transwell assays in control and OE33 miR‐196a overexpressing clones and quantification. Cells were seeded in the upper side of transwell chambers and after 72 h, cells migrated to the down side of the chamber were counted. Arrowheads indicate migrated cells. Scale bar, 100 μm. Data are mean + standard error of the mean of three independent experiments. **P < 0.01 and ***P < 0.001 for analysis of variance (ANOVA), plus Bonferroni post‐test.

To confirm that the EMT process allows tumor cells to increase their motility, scratch‐wound and transwell assays were performed in miR‐196a overexpressing cells. Control cells were able to heal 60–65% of the wound after 18 h, whereas both clones of cells overexpressing miR‐196a healed almost 100% (Fig. 2A,B). In addition, miR‐196a‐overexpressing cells showed an increased migration capability compared to control cells in transwell assays (Fig. 2C,D).

Finally, we overexpressed miR‐196a in the non‐transformed epithelial cell line Het1‐A. miR‐196a overexpression in Het1‐A cells (Fig. S2A) did not increase cell proliferation (Fig. S2B) and did not alter the expression levels of EMT markers *SNAIL1*, *CDH1*, and *VIMENTIN* (Fig. S2C–E).

Taken together, these results indicate that the overexpression of miR‐196a induces EMT and increases cell motility in OE33 cells, although it was unable to do so in normal esophagus epithelial cells.

### 3.2 miR‐196a effect is mediated by NFκB signaling

**Fig. 3:**
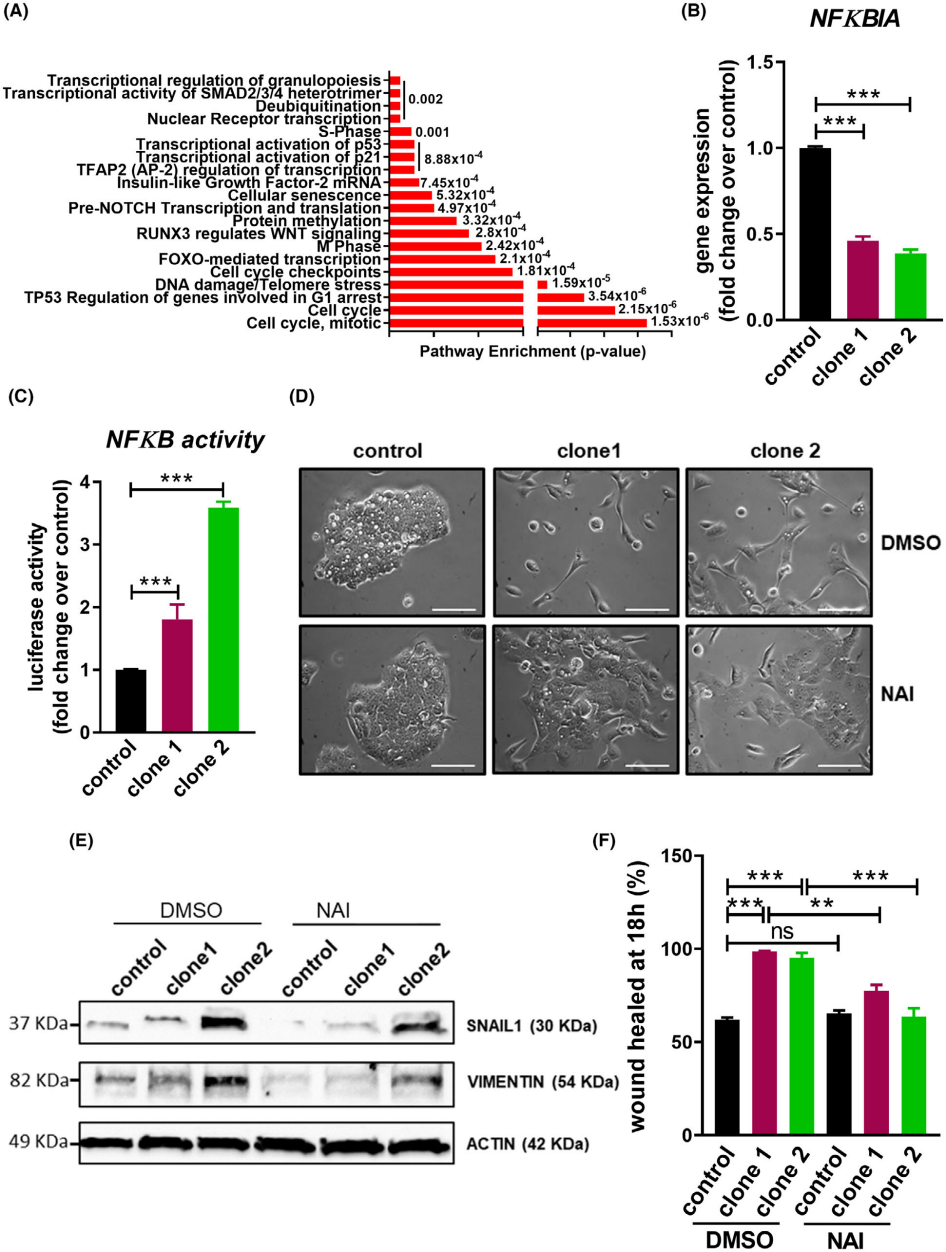
microRNA‐196a (miR‐196a) effects are mediated by the NFκB signaling pathway. (A) Pathway enrichment meta‐analysis of miRTarBase predicted targets for miR‐196a. (B) Gene expression of the miR‐196a validated target *NFKBIA* in control and OE33 miR‐196a overexpressing clones. (C) Activity of the NFκB pathway in control and OE33 miR‐196a clones. Cells were transfected with a plasmid containing 4xNFκB response elements controlling the expression of the downstream luciferase gene. After 48 h, protein extract was obtained and luciferase activity was quantified using the Dual‐Glo Luciferase Assay System. (D) Reversion of the mesenchymal phenotype of control and OE33 miR‐196a overexpressing clones after inhibition of NFκB. Cells were treated with 10 μm of NAI or DMSO as control for 18 h. Scale bar, 100 μm. (E) Western Blot images showing the inhibition of the increase in both SNAIL1 and VIMENTIN protein levels, and in cell motility (F) of control and OE33 miR‐196a overexpressing clones after inhibition of NFκB signaling using 10 μm of NAI or DMSO as control for 18 h. Data are mean + standard error of the mean of three independent experiments. **P < 0.01 and ***P < 0.001 for analysis of variance (ANOVA), plus Bonferroni post‐test. ns, not significant.

Next, we decided to decipher the molecular mechanisms by which miR‐196a exerts its effects in EAC cells by referring to the miR‐196a targets predicted by the miRTarBase database [15] to perform a pathway enrichment meta‐analysis using the Reactome Pathway Database (www.reactome.org) (Fig. 3A). The top pathways obtained in the analysis were related to cell cycle control, but miR‐196a did not increase proliferation compared to control (Fig. 1A). The next pathway obtained was the FOXO‐mediated transcription. The FoxO family of transcription factors translates various environmental stimuli into dynamic patterns of gene expression that influence a number of physiological and pathological processes, including cancer and aging [16]. Furthermore, *FOXO‐1* mRNA is a validated target of miR‐196a in cervical cancer, hepatocellular carcinoma, and lung cancer [17, 18, 19], and inhibits EMT in hepatocellular carcinoma [20]. *FOXO1* expression did not decrease in EAC cells transiently overexpressing miR‐196a or miR‐196b (Fig. S3A), suggesting that *FOXO1* mRNA is not a miR‐196a target in OE33 cells.

Among the validated targets of miR‐196a in the miRTarBase was the inhibitor of NFκB, alpha mRNA (*NFKBIA/IKBα*) [21]. NFκB signaling comprises a family of transcription factors that regulate, among other processes, tumorogenesis. In addition, NFκB signaling has been shown to induce EMT in gastric cancer [22] and has been related to EAC pathologies [23, 24]. Thus, we examined the expression of the NFKBIA gene in cells overexpressing miR‐196a and found that it decreased in both OE33 and OE19 stable cells and in OE33 cells transiently transfected with miR‐196a and miR‐196b (Fig. 3B and Fig. S3B,C). As expected, NFκB signaling was up‐regulated in EAC cells overexpressing miR‐196a and in those transiently transfected with miR‐196a and miR‐196b (Fig. 3C and Fig. S3D,E). In addition, treatment of OE33 stable cells overexpressing miR‐196a with the NFκB signaling inhibitor NAI (Fig. S3F) reverted the mesenchymal phenotype, reduced the expression of EMT markers *SNAIL1* and *VIMENTIN*, both at protein and mRNA levels, and decreased cell motility compared with cells treated with DMSO (Fig. 3D–F and Fig. S3G–I). All these observations demonstrate that the effects of overexpressing miR‐196a in EAC cells are, at least in part, mediated by NFκB signaling.

### 3.3 miR‐196a up‐regulates *TERT* expression, which reinforces NFκB signaling

**Fig. 4:**
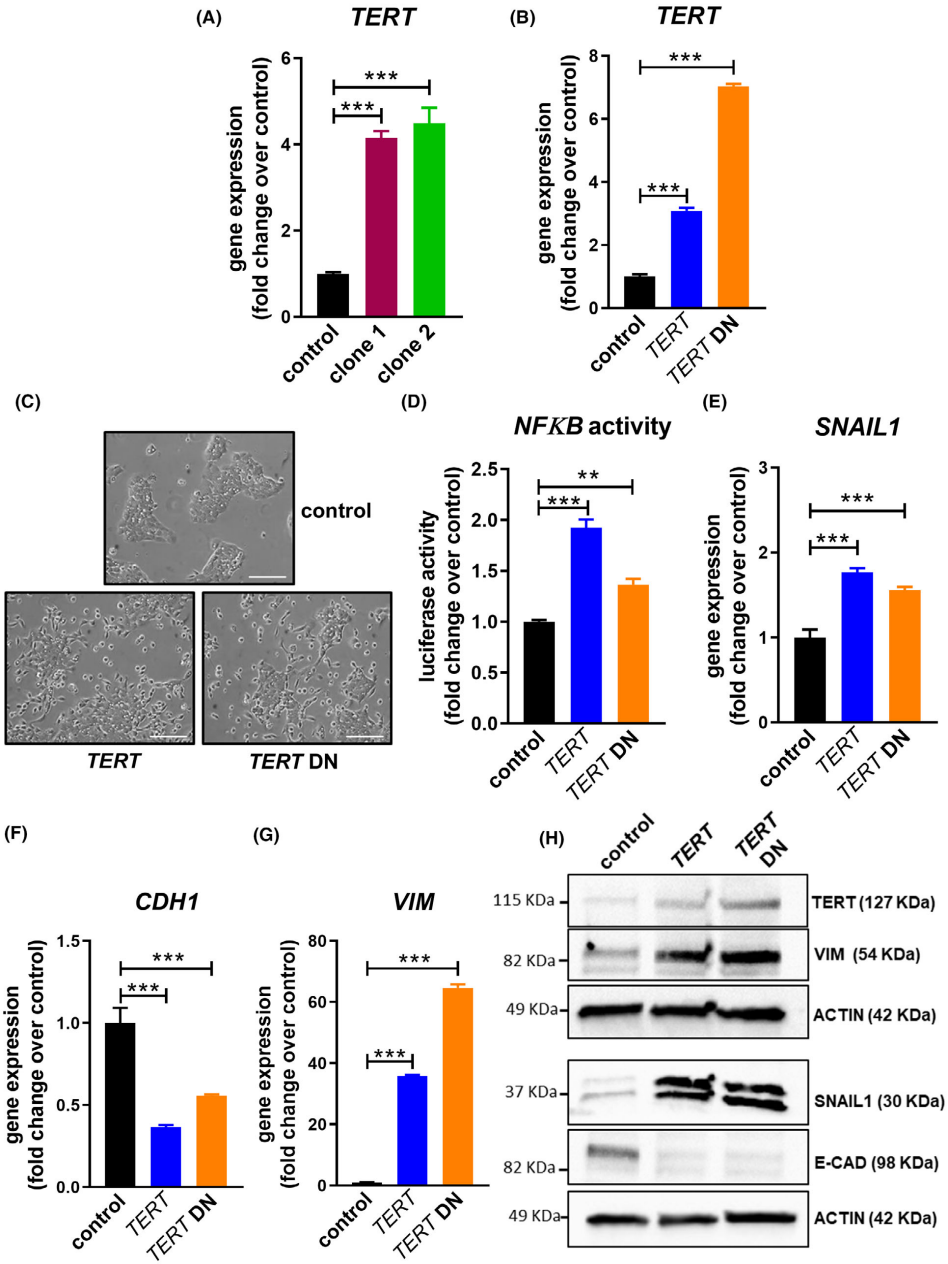
microRNA‐196a (miR‐196a) up‐regulates *TERT* expression, and TERT increases NFκB activity and produces epithelial‐to‐mesenchymal transition (EMT) in Esophageal Adenocarcinoma cells in a telomere lengthening‐independent manner. (A) Expression of *TERT*, the main component of the telomerase complex, by qPCR in control and OE33 mir196a overexpressing cells. (B) *TERT* expression in OE33 control and OE33 clones stably overexpressing *TERT* or a dominant negative form of *TERT* (*TERT DN*). (C) Phenotype switch in OE33 *TERT* and *TER‐DN* overexpressing clones. (D) *TERT* overexpression increases NFκB signaling activity. Control or OE33 clones stably overexpressing *TERT* or *TERT DN* were transfected with a plasmid containing 4xNFκB response elements controlling the expression of downstream luciferase gene. After 48 h, protein extract was obtained, and luciferase activity was quantified using the Dual‐Glo Luciferase Assay System. (E–H) qPCR and western blots of control and OE33 *TERT* and *TER‐DN* overexpressing clones showing the expression of EMT markers at both mRNA and protein levels. Scale bar, 100 μm. Data are mean + standard error of the mean of three independent experiments. **P < 0.01 and ***P < 0.001 for analysis of variance (ANOVA), plus Bonferroni post‐test.

**Fig. 5:**
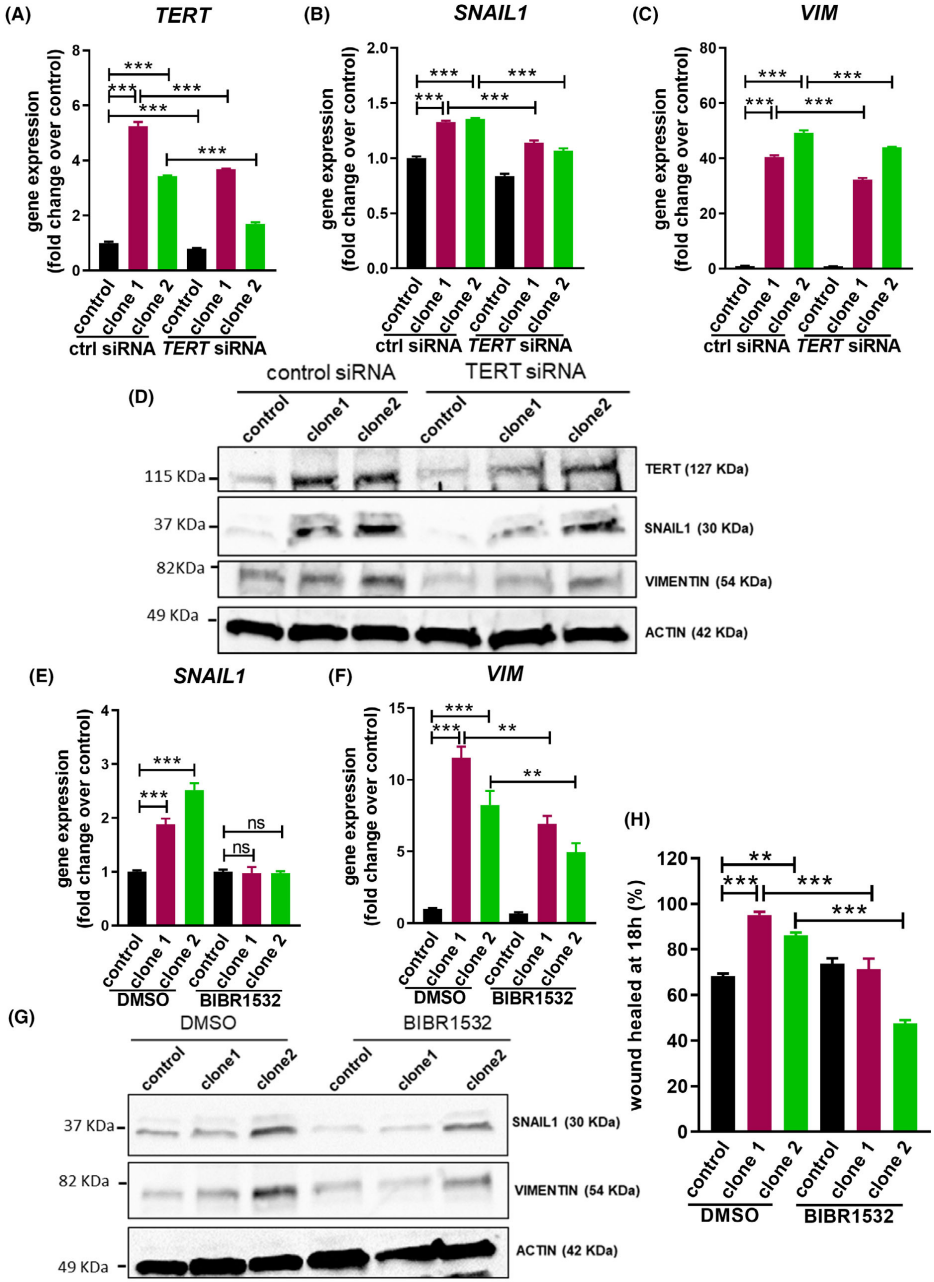
microRNA‐196a (miR‐196a) effects are mediated by TERT. (A–D) *TERT* siRNA interference reduces the expression of epithelial‐to‐mesenchymal transition markers (EMT) in miR‐196a overexpressing OE33 clones both at mRNA and protein levels. Cells were transfected using Lipofectamine 2000 with *TERT* siRNA or control siRNA (ctrl siRNA) and gene expression was analyzed after 48 h by qPCR (A–C). Protein expression in these cells was analyzed by western blot (D). (E–G) Reduction of miR‐196a‐mediated increase in the expression of EMT markers, both at mRNA and protein levels, upon TERT inhibition. Control cells and OE33 clones overexpressing miR‐196a were treated with 20 μm of BIBR1532 or DMSO as control for 18 h, and gene and protein expression were analyzed. (H) Reduction of miR‐196a‐mediated increase in cell motility upon TERT inhibition. Control cells and OE33 clones overexpressing miR‐196a were treated with 20 μm of BIBR1532 or DMSO as control for 18 h, and cell motility was analyzed by wound‐healing assay. Data are mean + standard error of the mean of three independent experiments. **P < 0.01 and ***P < 0.001 for analysis of variance (ANOVA), plus Bonferroni post‐test. ns, not significant.

Another pathway obtained in the Reactome meta‐analysis was the DNA damage/telomere stress pathway (Fig. 3A). Telomerase is a ribonucleoprotein complex that synthesizes telomeric repeats at the end of eukaryotic chromosomes [25]. Telomerase is essential for maintaining pools of proliferating cells in adulthood, including tumor cells, and telomerase expression is reactivated in around 85% of all cancers [26]. Consequently, we evaluated the expression of telomerase reverse‐transcriptase (*TERT*) and the telomerase RNA component (*TERC*), the main components of the telomerase complex, in cells overexpressing miR‐196a. Interestingly, while *TERC* expression was reduced in OE33 miR‐196a overexpressing cells, *TERT* expression increased 4‐fold in both OE33 and OE19 cells (Fig. S4A,B and Fig. 4A). The down‐regulation of *TERC* levels by siRNA transfection in parental OE33 cells did not increase *SNAIL1* or *VIMENTIN* mRNA amounts (Fig. S4C–E). However, OE33 cells stably overexpressing *TERT* (Fig. 4B,I, upper panel) presented a phenotype switch to a mesenchymal shape, increased NFκB signaling, up‐regulated the levels of *SNAIL1* and *VIMENTIN*, both at protein and mRNA levels, and decreased the expression of *CDH1* compared with control cells. Strikingly, the same effects were observed in OE33 cells stably overexpressing a dominant negative form of telomerase (TERT‐DN), which is devoid of the ability to perform telomere lengthening (Fig. 4C–H and Fig. S4F). Accordingly, the down‐regulation of *TERT* in miR‐196a overexpressing cells using siRNA transfection reduced *SNAIL1* and *VIMENTIN* expression, both at protein and mRNA levels, compared with the control (Fig. 5A–D). Furthermore, the treatment of these cells with the dual, telomerase activity and TERT non‐canonical function inhibitor BIBR1532 [27] (Fig. S5A) reverted the mesenchymal phenotype (Fig. S5B) and also resulted in a decrease of *SNAIL1* and *VIMENTIN* mRNA and protein levels, as well as in cell motility, when compared with control cells treated with DMSO (Fig. 5E–H and Fig. S5C). Interestingly, the up‐regulation of *TERT* expression in miR‐196a overexpressing cells is not dependent on NFκB signaling, since the treatment with the inhibitor NAI did not decrease the levels of *TERT* mRNA (Fig. S4G). Overall, these results demonstrate that miR‐196a overexpression, at least in part, increases *TERT* expression independently of NFκB signaling and that TERT then reinforces the NFκB signaling in a telomere lengthening‐independent manner.

### 3.4 The effects of miR‐196a depend on c‐MYC protein

**Fig. 6:**
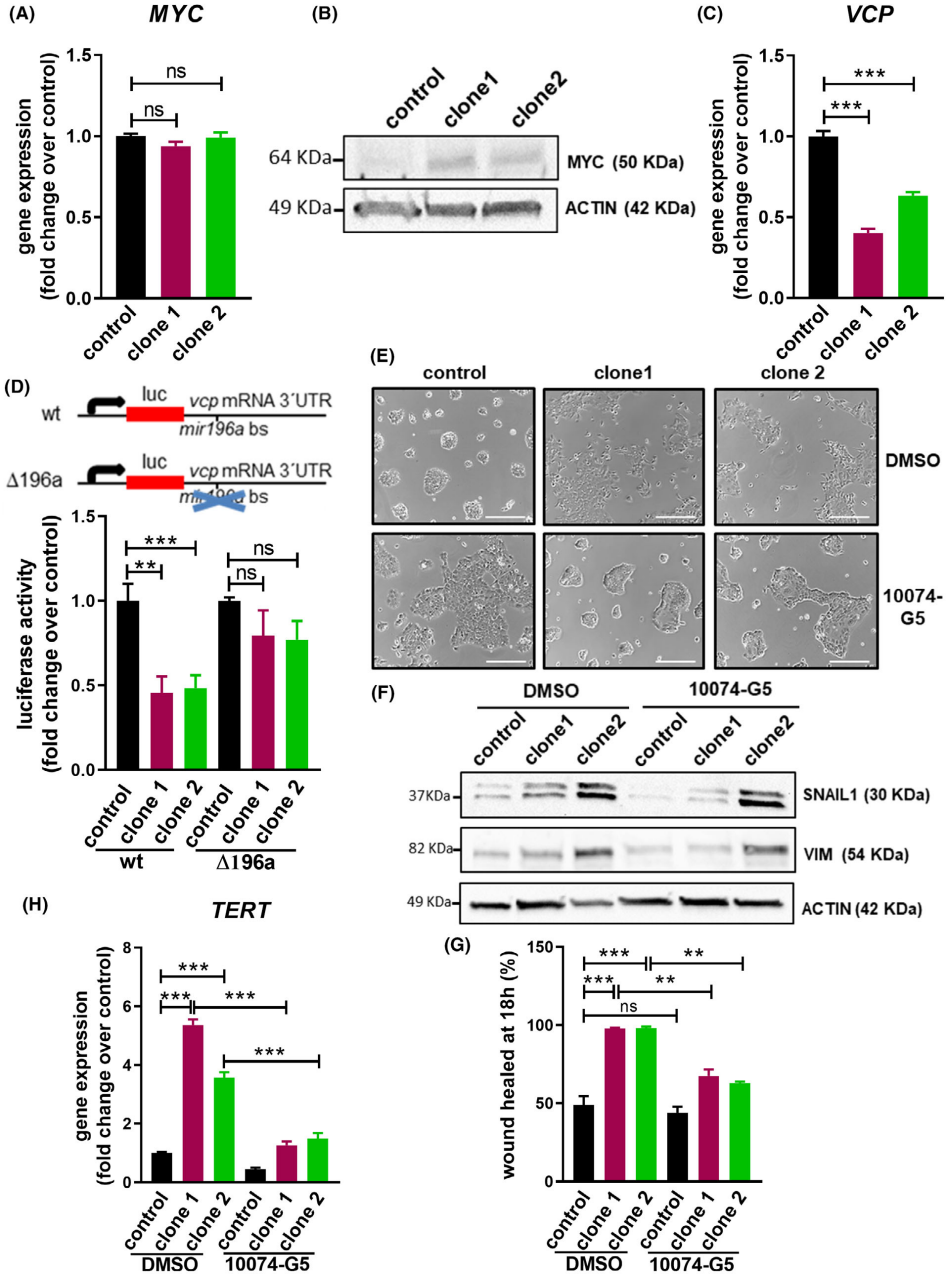
microRNA‐196a (miR‐196a) induces epithelial‐to‐mesenchymal transition (EMT) and cell motility via c‐MYC protein accumulation. (A) *c‐MYC* mRNA levels in control cells and OE33 miR‐196a overexpressing clones. (B) Western blot of c‐MYC protein in control cells and OE33 miR‐196a overexpressing clones. (C) *VCP* mRNA levels in control cells and OE33 miR‐196a overexpressing clones. (D) Luciferase reporter assay in control and miR‐196a overexpressing OE33 clones. Wild type (wt) 3′ untranslated region of *VCP* mRNA (*vcp* mRNA 3′ UTR) or deletion of putative mir‐196a binding sequences (Δ196a) were cloned downstream of the *luciferase* (luc) gene. Luciferase constructs were then transfected in control cells or OE33 miR‐196a overexpressing clones, and luciferase activity was quantified using the Dual‐Glo Luciferase Assay System after 48 h. (E) Reversal of the mesenchymal phenotype of OE33 miR‐196a overexpressing clones after inhibition of c‐MYC activity. Cells were plated in six well plates and treated with 50 μm of 10 074‐G5 or DMSO as control for 18 h. Scale bar, 100 μm. (F) Western blot of SNAIL1 and VIMENTIN protein levels in control cells or OE33 miR‐196a overexpressing clones, and *TERT* mRNA levels in control cells and OE33 miR‐196a overexpressing clones upon c‐MYC inhibition (H). Cells were plated in six well plates and treated with 50 μm of 10 074‐G5 or DMSO as control for 18 h. Data are mean + standard error of the mean of three independent experiments. **P < 0.01 and ***P < 0.001 for analysis of variance (ANOVA), plus Bonferroni post‐test. ns, not significant.

The most typical way in which telomerase expression is up‐regulated in tumors is through the overexpression of transcriptional factor *MYC* [28]. We therefore assessed the levels of *MYC* mRNA in OE33 cells overexpressing miR‐196a and found that they were unaffected. However, western blot experiments identified an accumulation of the protein when the level of miR196a was increased (Fig. 6A,B). The level and activity of c‐MYC protein are mainly controlled and regulated by ubiquitination and deubiquitination and are mediated by a number of ubiquitin ligases and deubiquitinating enzymes [29]. In addition, protein deubiquitination is a molecular pathway obtained in the meta‐analysis performed for miR‐196a targets (Fig. 3A). Putative miR‐196a target genes grouped in the pathway were the ubiquitin‐conjugating enzyme E2 C (*UBE2C*), ubiquitin‐conjugating enzyme E2 Z (*UBE2Z*), the peroxisomal membrane protein 13 (*PEX13*) and valosin‐containing protein (*VCP*). Only *VCP* expression showed an inverse correlation with miR‐196a expression in a cohort of 109 patients with esophageal tumors from The Cancer Genome Atlas (TCGA) study (Fig. S6A–D), suggesting that *VCP* mRNA might be a relevant miR‐196a target in esophageal tumor patients. Furthermore, VCP has been shown to regulate the levels and function of c‐MYC protein through ubiquitination [30]. Interestingly, both OE33 and OE19 clones overexpressing miR‐196a showed a decrease in *VCP* mRNA levels (Fig. 6C and Fig. S7A). Luciferase reporter assays using the 3′ UTR fragment of VCP mRNA, harboring the predicted mir196a binding site (mir196a bs), showed a decrease in luciferase mRNA stability when mir196a was overexpressed. This effect did not happen when a luciferase construct with a deletion of the mir196a bs in the VCP 3′ UTR was used. These results consistently indicated that *VCP* is a target of miR‐196a in OE33 cells (Fig. 6D). Finally, to confirm that the miR‐196a effects in EAC cells were mediated by c‐MYC, cells overexpressing miR‐196a were treated with the c‐MYC/MAX dimerization inhibitor 10074‐G5. It was found that c‐MYC inhibition reversed the mesenchymal shape, reduced the expression of EMT markers at both protein and mRNA levels, and *TERT*, and decreased cell motility compared with control cells treated with DMSO (Fig. 6E–H and Fig. S7B–D). Altogether, these results indicate that c‐MYC protein is accumulated in cells overexpressing miR‐196a, increases *TERT* expression, and mediates, at least in part, the effects of miR‐196a.

### 3.5 The C‐MYC/TERT/NFκB signaling axis is increased in Barrett's esophagus patients with high miR196a expression

**Fig. 7:**
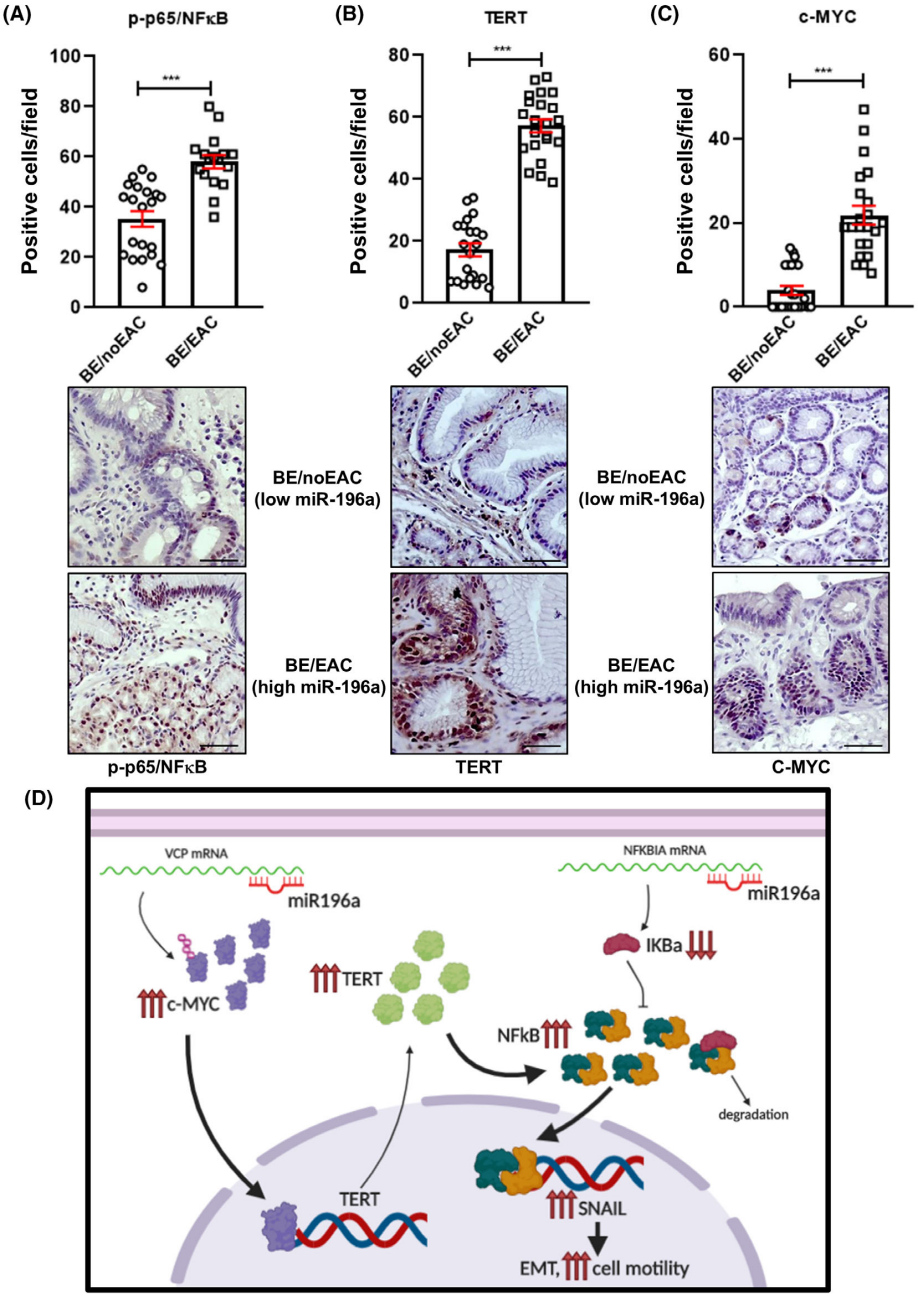
The MYC/TERT/NFκB axis is hyperactive in Barrett Esophagus (BE) patients with high miR‐196a expression. Representative images of immunohistochemistries for NFκB (A), TERT (B) and c‐MYC (C) in BE biopsies of patients with high expression of miR‐196a, who progressed to Esophagus Adenocarcinoma (BE/EAC), compared with samples from BE patients with low miR‐196a expression levels who did not progress (BE/noEAC), and their quantification. Each dot represents a microscope field count for *n* = 6 patients in the BE/noEAC group and *n* = 5 patients in the BE/EAC group, with at least three counts per patient sample. Scale bars, 50 μm. Data are mean + standard error of the mean. ***P < 0.001 for the Mann–Whitney test. (D) Model depicting the mechanism of action of miR‐196a in EAC cells and patients.

Next, we evaluated whether the molecular mechanisms mediating the effects of miR‐196a unveiled in EAC cells also occurred in BE/EAC patients with high miR‐196a expression. Interestingly, the expression of *SNAIL1* and *TERT* directly correlated with the expression of miR‐196a in the TCGA cohort of 109 patients with esophageal cancer (Fig. S8A,B). It was consistently found that the expression of the miR‐196a target *NFKBIA* inversely correlated with miR‐196a expression in the same cohort of patients (Fig. S8C). *SNAIL1* and *TERT* expression also showed a direct correlation in these patients (Fig. S8D). Finally, we assayed by immunohistochemistry the protein levels in BE patient samples with high miR196a levels that eventually developed EAC and compared the findings with samples from patients with low miR‐196a expression that did not progress. The expression of mir196a in all the patient samples used here was previously analyzed and was found to be higher in those patients that progressed to EAC compared to those who did not [12]. Very strikingly, NFκB signaling, TERT, and c‐MYC showed higher levels of expression and activity in samples of patients that developed EAC than those that did not (Fig. 7A–C and Fig. S8F). The results strongly suggest that the action mechanism described for miR‐196a is operating in patients and might be responsible, at least in part, for disease progression in BE patients with high levels of miR‐196a.

## 4 Discussion

This study set out to answer the question of whether miR‐192, miR‐194, miR‐196a, and miR‐196b, which were overexpressed in BE samples of patients that developed EAC compared to samples of patients that did not develop EAC [12], might also be drivers of the process of tumorogenesis. Family members of miR‐196 (a and b) differ only in one nucleotide in their target‐binding sequence, although they are produced from two different loci and have the same predicted targets in the miRTarBase database [15]. In our study, neither miR‐196a nor b overexpression had any effect on OE33 cell proliferation, although they induced an evident phenotypic change to a mesenchymal shape, as in epithelial‐to‐mesenchymal transition (EMT). This was further confirmed in both OE19 and OE33 stable cells overexpressing miR‐196a, when the EMT process was accompanied by an increase in cell motility. Indeed, miR‐196a has been seen to be involved in the onset and progression of a number of cancers, participating in tumor proliferation and in migration/invasion [31]. In esophageal squamous cell carcinoma, miR‐196a has been shown to inhibit tumor migration and invasion [32], while in EAC the available data do not establish whether miR‐196a regulates proliferation and/or migration/invasion [33, 34]. The EMT process is a prerequisite for tumor invasion and is regulated by several transcription factors, including SNAIL1/2, ZEB1/2, and TWIST [14]. The miR200 family of miRNAs is up‐regulated in BE and suppressed in EAC and is known to regulate EMT through ZEB1/2 inhibition, but the role of these miRNAs in EAC pathology is poorly defined [10, 11]. In this study, however, we show that miR‐196a may fuel tumor progression by increasing the migration capacity of EAC cells, rather than by promoting cell proliferation. Furthermore, to the best of our knowledge, miR‐196a is the first microRNA clearly shown to induce the EMT process in EAC cells. Curiously, overexpression of miR‐196a in the normal esophagus epithelial cell line Het‐1A did not alter cell proliferation nor promote EMT, suggesting that high levels of mir196a would not be sufficient to induce transformation/aggressiveness in these cells.

Although not expressed in normal squamous esophageal epithelium, NFκB signaling is high in BE and EAC tissues [24] and correlates with BE progression to EAC [23, 35]. The miR‐21, miR‐130b, and miR‐181b are up‐regulated in BE and positively regulate NFκB signaling in other models, although mechanistic studies into the roles played by these miRNAs in regulating the pathway in the particular case of BE/EAC are lacking [36, 37]. However, we have shown that the effects of miR‐196a in EAC cells are mediated by an increase in the activity of the NFκB signaling pathway, since miR‐196a reduced NFκB inhibitor alpha (*NFKBIA*) mRNA levels. Thus, *NFKBIA* mRNA is probably a target of miR‐196a in EAC, as it is in pancreatic cancer [21] and glioblastoma [38]. NFκB signaling mediates the induction of EMT in gastric cancer [22] and breast cancer [39]. In addition, the NFκB subunit REL‐A has been shown to directly regulate EMT by binding to the promoter of *SNAIL1* in airway epithelial cells [40]. Therefore, we hypothesize that the increase in the active REL‐A subunit of NFκB in the nucleus of EAC cells overexpressing miR‐196a is directly responsible for an increase in *SNAIL1* expression and the induction of EMT, although it cannot be ruled out that this occurs via an indirect function.

Telomerase reactivation has been found in around 85% of all human cancers, where it allows cancer cells to prevent telomere attrition and replicative senescence since cells divide indefinitely. We found that miR‐196a up‐regulates *TERT* expression. A high degree of methylation of the *TERT* promoter was shown to correlate with a higher risk of BE progression to EAC, although the same study did not show that *TERT* levels were actually reduced [41]. On the contrary, we clearly show here that *TERT* overexpression positively regulates oncogenic processes, such as EMT in EAC. TERT has been shown to form a complex with ZEB1 to directly mediate EMT by binding to the *CDH1* promoter [42], but we show that EMT is mediated by SNAIL1, and that *TERT* overexpression in OE33 cells induces an increase in NFκB signaling and EMT. Thus, an increase in *TERT* in cells overexpressing miR‐196a very likely reinforces NFκB signaling which, in turn, mediates EMT. Very interestingly, a dominant negative form of TERT lacking telomerase activity produces the same effects as *wt* TERT in EAC cells. This suggests that high levels of TERT may be involved in EAC oncogenesis, independently of telomere elongation activity, as reported for other types of cancer [43].

Using comparative genomic hybridization techniques, two studies at the end of the last century showed that copy number alterations of the MYC proto‐oncogene were involved in BE/EAC pathogenesis [44, 45], but the precise role of these amplifications was not defined. Here we show that miR‐196a controls MYC protein levels post‐transcriptionally in EAC cells, very likely by controlling the levels of *VCP*, a protein involved in the ubiquitin pathway of protein degradation by the proteasome, and which has been related to MYC function. Finally, and very strikingly, we show that the MYC/TERT/NFκB molecular axis, described as mediating miR196a oncogenic activity in EAC cells, is also operating in BE patients and, most importantly, is hyperactive by the time of biopsy extraction for the diagnosis of BE. Patients with high levels of miR‐196a, with a higher risk of progression to EAC, show higher amounts of c‐MYC, TERT, and active p65NFκB subunit compared with BE samples from patients with low miR196a expression levels and a low risk of progression. These results indicate that this molecular pathway could be used to design new therapeutic interventions to prevent pathway activation. More particularly, the MYC oncogene has historically been recognized as a ‘most wanted’ target for cancer therapy, since it is deregulated in most, perhaps all, human cancers. Therefore, MYC could be a very promising target for therapeutic intervention in BE/EAC progression. Very interestingly, a MYC dominant negative peptide, *Omomyc*, has shown remarkable anti‐cancer properties in a wide range of tumor types and now is starting to be applied in clinical practice [46, 47]. Moreover, the levels of miR196a, c‐MYC, TERT, and NFκB in biopsies might be used as prognostic markers to identify BE patients with a high risk of developing EAC.

## 5 Conclusion

In conclusion, a molecular mechanism that is activated in EAC cells overexpressing miR‐196a was identified (Fig. 7D). On the one hand, miR‐196a would reduce the levels of *NFKBIA* mRNA, resulting in higher NFκB signaling and increasing the induction of EMT and cell motility. At the same time, miR‐196a would target *VCP* mRNA, which would result in an accumulation of MYC, leading to an increase in *TERT* expression and finally, reinforcing NFκB signaling. It was also seen that the MYC/TERT/NFκB signaling axis was in addition hyperactive in BE samples of patients that progress to EAC.

## Conflict of interest

The authors declare no conflict of interest.

## Author contributions

JG‐C, LFMH, and MLC conceived the study; JG‐C, CMM‐C, MLC, and LFMH designed research; JG‐C, CMM‐C, and MB‐G performed research; JG‐C, CMM‐C, MB‐G, VM, DRA, PP, AO, LFMH, and MLC analyzed data, and JG‐C, LFMH, and MLC wrote the manuscript.

## Peer review

The peer review history for this article is available at https://www.webofscience.com/api/gateway/wos/peer‐review/10.1002/1878‐0261.70048.

## Supporting information


**Fig. S1.** microRNA (miRNA) 196 family induces a phenotype switch in OE33 cells and increased expression of epithelial‐to‐mesenchymal transition markers in OE19 cells.
**Fig. S2.** Overexpression of miR‐196a in non‐transformed esophagus epithelial Het‐1A cells does not induce aggressiveness traits.
**Fig. S3.** miR‐196a effects are mediated by NFκB signaling pathway.
**Fig. S4.** miR‐196a effects are not mediated by TERC.
**Fig. S5.** miR‐196a effects are mediated by *TERT*.
**Fig. S6.** miR‐196a effects are mediated via c‐MYC.
**Fig. S7.** miR‐196a effects are mediated via c‐MYC.
**Fig. S8.** MYC/TERT/NFκB axis is hyperactive in BE patients with high risk of developing EAC.
**Table S1.** Primers used in this study.

## Data Availability

The data that support the findings of this study are available from the corresponding authors (jesus.garcia@ffis.es and marial.cayuela@carm.es) upon reasonable request.

## References

[1] Caspa Gokulan R , Garcia‐Buitrago MT , Zaika AI . From genetics to signaling pathways: molecular pathogenesis of esophageal adenocarcinoma. Biochim Biophys Acta Rev Cancer. 2019;1872:37–48. 31152823 10.1016/j.bbcan.2019.05.003 PMC6692203

[2] Jiang M , Li H , Zhang Y , Yang Y , Lu R , Liu K , et al. Transitional basal cells at the squamous‐columnar junction generate Barrett's oesophagus. Nature. 2017;550:529–533. 29019984 10.1038/nature24269 PMC5831195

[3] Sharma P . Clinical practice. Barrett's esophagus. N Engl J Med. 2009;361:2548–2556. 20032324 10.1056/NEJMcp0902173

[4] Martinez de Haro L , Ortiz A , Parrilla P , Munitiz V , Molina J , Bermejo J , et al. A Intestinal metaplasia in patients with columnar lined esophagus is associated with high levels of duodenogastroesophageal reflux. Ann Surg. 2001;233:34–38. 11141222 10.1097/00000658-200101000-00006 PMC1421163

[5] Codipilly DC , Chandar AK , Singh S , Wani S , Shaheen NJ , Inadomi JM , et al. The effect of endoscopic surveillance in patients with Barrett's esophagus: a systematic review and meta‐analysis. Gastroenterology. 2018;154:2068–2086. 29458154 10.1053/j.gastro.2018.02.022 PMC5985204

[6] Qiao Y , Hyder A , Bae SJ , Zarin W , O'Neill TJ , Marcon NE , et al. Surveillance in patients with Barrett's esophagus for early detection of esophageal adenocarcinoma: a systematic review and meta‐analysis. Clin Transl Gastroenterol. 2015;6:e131. 10.1038/ctg.2015.58 PMC4816094 26658838

[7] Bartel DP . MicroRNAs: genomics, biogenesis, mechanism, and function. Cell. 2004;116:281–297. 14744438 10.1016/s0092-8674(04)00045-5

[8] Schwarzenbach H , Nishida N , Calin GA , Pantel K . Clinical relevance of circulating cell‐free microRNAs in cancer. Nat Rev Clin Oncol. 2014;11:145–156. 24492836 10.1038/nrclinonc.2014.5

[9] Clark RJ , Craig MP , Agrawal S , Kadakia M . microRNA involvement in the onset and progression of Barrett's esophagus: a systematic review. Oncotarget. 2018;9:8179–8196. 29487725 10.18632/oncotarget.24145 PMC5814292

[10] Gregory PA , Bert AG , Paterson EL , Barry SC , Tsykin A , Farshid G , et al. The miR‐200 family and miR‐205 regulate epithelial to mesenchymal transition by targeting ZEB1 and SIP1. Nat Cell Biol. 2008;10:593–601. 18376396 10.1038/ncb1722

[11] Smith CM , Watson DI , Leong MP , Mayne GC , Michael MZ , Wijnhoven BP , et al. miR‐200 family expression is downregulated upon neoplastic progression of Barrett's esophagus. World J Gastroenterol. 2011;17:1036–1044. 21448356 10.3748/wjg.v17.i8.1036 PMC3057147

[12] Revilla‐Nuin B , Parrilla P , Lozano JJ , de Haro LF , Ortiz A , Martínez C , et al. Predictive value of MicroRNAs in the progression of barrett esophagus to adenocarcinoma in a long‐term follow‐up study. Ann Surg. 2013;257(5):886–893. 10.1097/SLA.0b013e31826ddba6 23059500

[13] Rockett JC , Larkin K , Darnton SJ , Morris AG , Matthews HR . Five newly established oesophageal carcinoma cell lines: phenotypic and immunological characterization. Br J Cancer. 1997;75:258–263. 9010035 10.1038/bjc.1997.42 PMC2063267

[14] Dongre A , Weinberg RA . New insights into the mechanisms of epithelial‐mesenchymal transition and implications for cancer. Nat Rev Mol Cell Biol. 2019;20:69–84. 30459476 10.1038/s41580-018-0080-4

[15] Huang HY , Lin YC , Li J , Huang KY , Shrestha S , Hong HC , et al. miRTarBase 2020: updates to the experimentally validated microRNA‐target interaction database. Nucleic Acids Res. 2020;48(D1):D148–D154. 10.1093/nar/gkz896 31647101 PMC7145596

[16] Kim SY , Ko YS , Park J , Choi Y , Park JW , Kim Y , et al. Forkhead transcription factor FOXO1 inhibits angiogenesis in gastric cancer in relation to SIRT1. Cancer Res Treat. 2016;48:345–354. 25761483 10.4143/crt.2014.247 PMC4720104

[17] Guerriero I , D'Angelo D , Pallante P , Santos M , Scrima M , Malanga D , et al. Analysis of miRNA profiles identified miR‐196a as a crucial mediator of aberrant PI3K/AKT signaling in lung cancer cells. Oncotarget. 2017;8(12):19172–19191. 10.18632/oncotarget.13432 27880728 PMC5386676

[18] Hou T , Ou J , Zhao X , Huang X , Huang Y , Zhang Y . MicroRNA‐196a promotes cervical cancer proliferation through the regulation of FOXO1 and p27Kip1. Br J Cancer. 2014;110:1260–1268. 24423924 10.1038/bjc.2013.829 PMC3950858

[19] Xu H , Li G , Yue Z , Li C . HCV core protein‐induced upregulation of microRNA‐196a promotes aberrant proliferation in hepatocellular carcinoma by targeting FOXO1. Mol Med Rep. 2016;13:5223–5229. 27108614 10.3892/mmr.2016.5159

[20] Dong T , Zhang Y , Chen Y , Liu P , An T , Zhang J , et al. FOXO1 inhibits the invasion and metastasis of hepatocellular carcinoma by reversing ZEB2‐induced epithelial‐mesenchymal transition. Oncotarget. 2017;8:1703–1713. 27924058 10.18632/oncotarget.13786 PMC5352090

[21] Huang F , Tang J , Zhuang X , Zhuang Y , Cheng W , Chen W , et al. MiR‐196a promotes pancreatic cancer progression by targeting nuclear factor kappa‐B‐inhibitor alpha. PLoS One. 2014;9:e87897. 24504166 10.1371/journal.pone.0087897 PMC3913664

[22] Li J , Deng Z , Wang Z , Wang D , Zhang L , Su Q , et al. Zipper‐interacting protein kinase promotes epithelial‐mesenchymal transition, invasion and metastasis through AKT and NF‐kB signaling and is associated with metastasis and poor prognosis in gastric cancer patients. Oncotarget. 2015;6:8323–8338. 25831050 10.18632/oncotarget.3200 PMC4480755

[23] McAdam E , Haboubi HN , Griffiths AP , Baxter JN , Spencer‐Harty S , Davies C , et al. Reflux composition influences the level of NF‐κB activation and upstream kinase preference in oesophageal adenocarcinoma cells. Int J Cancer. 2015;136:527–535. 24931696 10.1002/ijc.29029

[24] O'Riordan JM , Abdel‐latif MM , Ravi N , McNamara D , Byrne PJ , McDonald GS , et al. Proinflammatory cytokine and nuclear factor kappa‐B expression along the inflammation‐metaplasia‐dysplasia‐adenocarcinoma sequence in the esophagus. Am J Gastroenterol. 2005;100(6):1257–1264. 10.1111/j.1572-0241.2005.41338.x 15929754

[25] Blackburn EH . Telomeres and telomerase: their mechanisms of action and the effects of altering their functions. FEBS Lett. 2005;579:859–862. 15680963 10.1016/j.febslet.2004.11.036

[26] Akincilar SC , Unal B , Tergaonkar V . Reactivation of telomerase in cancer. Cell Mol Life Sci. 2016;73:1659–1670. 26846696 10.1007/s00018-016-2146-9 PMC4805692

[27] Bernabé‐García M , Martínez‐Balsalobre E , García‐Moreno D , García‐Castillo J , Revilla‐Nuin B , Blanco‐Alcaina E , et al. Telomerase reverse transcriptase (TERT) activates transcription of miR500A to inhibit hedgehog signaling and promote cell invasiveness. Mol Oncol. 2021;15:1118–1134. 10.1002/1878-0261.12943 PMC8253104 33713376

[28] Yuan X , Larsson C , Xu D . Mechanisms underlying the activation of TERT transcription and telomerase activity in human cancer: old actors and new players. Oncogene. 2019;38:6172–6183. 31285550 10.1038/s41388-019-0872-9 PMC6756069

[29] Farrell AS , Sears RC . MYC degradation. Cold Spring Harb Perspect Med. 2014;4:a014365. 24591536 10.1101/cshperspect.a014365 PMC3935390

[30] Heidelberger JB , Voigt A , Borisova ME , Petrosino G , Ruf S , Wagner SA , et al. Proteomic profiling of VCP substrates links VCP to K6‐linked ubiquitylation and c‐Myc function. EMBO Rep. 2018;19:e44754. 29467282 10.15252/embr.201744754 PMC5891417

[31] Chen ZY , Chen X , Wang ZX . The role of microRNA‐196a in tumorigenesis, tumor progression, and prognosis. Tumour Biol. 2016;37:15457–15466. 27752997 10.1007/s13277-016-5430-2

[32] Wang K , Li J , Guo H , Xu X , Xiong G , Guan X , et al. MiR‐196a binding‐site SNP regulates RAP1A expression contributing to esophageal squamous cell carcinoma risk and metastasis. Carcinogenesis. 2012;33:2147–2154. 22859270 10.1093/carcin/bgs259

[33] Luthra R , Singh RR , Luthra MG , Li YX , Hannah C , Romans AM , et al. MicroRNA‐196a targets annexin A1: a microRNA‐mediated mechanism of annexin A1 downregulation in cancers. Oncogene. 2008;27:6667–6678. 18663355 10.1038/onc.2008.256

[34] Maru DM , Singh RR , Hannah C , Albarracin CT , Li YX , Abraham R , et al. MicroRNA‐196a is a potential marker of progression during Barrett's metaplasia‐dysplasia‐invasive adenocarcinoma sequence in esophagus. Am J Pathol. 2009;174:1940–1948. 19342367 10.2353/ajpath.2009.080718 PMC2671281

[35] Abdel‐Latif MM , O'Riordan J , Windle HJ , Carton E , Ravi N , Kelleher D , et al. NF‐kappaB activation in esophageal adenocarcinoma: relationship to Barrett's metaplasia, survival, and response to neoadjuvant chemoradiotherapy. Ann Surg. 2004;239:491–500. 15024310 10.1097/01.sla.0000118751.95179.c6 PMC1356254

[36] Iliopoulos D , Jaeger SA , Hirsch HA , Bulyk ML , Struhl K . STAT3 activation of miR‐21 and miR‐181b‐1 via PTEN and CYLD are part of the epigenetic switch linking inflammation to cancer. Mol Cell. 2010;39:493–506. 20797623 10.1016/j.molcel.2010.07.023 PMC2929389

[37] Wang Y , Mao G , Lv Y , Huang Q , Wang G . MicroRNA‐181b stimulates inflammation via the nuclear factor‐κB signaling pathway. Exp Ther Med. 2015;10:1584–1590. 26622531 10.3892/etm.2015.2702 PMC4578103

[38] Yang G , Han D , Chen X , Zhang D , Wang L , Shi C , et al. MiR‐196a exerts its oncogenic effect in glioblastoma multiforme by inhibition of IκBα both in vitro and in vivo. Neuro Oncol. 2014;16:652–661. 24463357 10.1093/neuonc/not307 PMC3984554

[39] Storci G , Sansone P , Mari S , D'Uva G , Tavolari S , Guarnieri T , et al. TNFalpha up‐regulates SLUG via the NF‐kappaB/HIF1alpha axis, which imparts breast cancer cells with a stem cell‐like phenotype. J Cell Physiol. 2010;225:682–691. 20509143 10.1002/jcp.22264 PMC2939957

[40] Tian B , Widen SG , Yang J , Wood TG , Kudlicki A , Zhao , et al. The NFκB subunit RELA is a master transcriptional regulator of the committed epithelial‐mesenchymal transition in airway epithelial cells. J Biol Chem. 2018;293:16528–16545. 30166344 10.1074/jbc.RA118.003662 PMC6200927

[41] Clément G , Braunschweig R , Pasquier N , Bosman FT , Benhattar J . Methylation of APC, TIMP3, and TERT: a new predictive marker to distinguish Barrett's oesophagus patients at risk for malignant transformation. J Pathol. 2006;208:100–107. 16278815 10.1002/path.1884

[42] Qin Y , Tang B , Hu CJ , Xiao YF , Xie R , Yong X , et al. An hTERT/ZEB1 complex directly regulates E‐cadherin to promote epithelial‐to‐mesenchymal transition (EMT) in colorectal cancer. Oncotarget. 2016;7:351–361. 26540342 10.18632/oncotarget.5968 PMC4808003

[43] Li Y , Tergaonkar V . Noncanonical functions of telomerase: implications in telomerase‐targeted cancer therapies. Cancer Res. 2014;74:1639–1644. 24599132 10.1158/0008-5472.CAN-13-3568

[44] Moskaluk CA , Hu J , Perlman EJ . Comparative genomic hybridization of esophageal and gastroesophageal adenocarcinomas shows consensus areas of DNA gain and loss. Genes Chromosomes Cancer. 1998;22:305–311. 9669668

[45] van Dekken H , Geelen E , Dinjens WN , Wijnhoven BP , Tilanus HW , Tanke HJ , et al. Comparative genomic hybridization of cancer of the gastroesophageal junction: deletion of 14Q31‐32.1 discriminates between esophageal (Barrett's) and gastric cardia adenocarcinomas. Cancer Res. 1999;59:748–752. 9973227

[46] Massó‐Vallés D , Soucek L . Blocking Myc to treat cancer: reflecting on two decades of Omomyc. Cells. 2020;9:883. 32260326 10.3390/cells9040883 PMC7226798

[47] Whitfield JR , Beaulieu ME , Soucek L . Strategies to inhibit Myc and their clinical applicability. Front Cell Dev Biol. 2017;5:10. 28280720 10.3389/fcell.2017.00010 PMC5322154

