# Supplementary material for: MicroRNA 196a contributes to the aggressiveness of esophageal adenocarcinoma through the MYC/TERT/NFκB axis
Source: Mol Oncol. 2025 Sep 16;19(11):3305–24. doi: 10.1002/1878-0261.70048 (PMC12591326; doi:10.1002/1878-0261.70048)
Supplement: Supplementary file 1 — Fig. S1. microRNA (miRNA) 196 family induces a phenotype switch in OE33 cells and increased expression of epithelial‐to‐mesenchymal transition markers in OE19 cells. Fig. S2. Overexpression of miR‐196a in non‐transformed esophagus epithelial Het‐1A cells does not induce aggressiveness traits. Fig. S3. miR‐196a effects are mediated by NFκB signaling pathway. Fig. S4. miR‐196a effects are not mediated by TERC. Fig. S5. miR‐196a effects are mediated by TERT. Fig. S6. miR‐196a effects are mediated via c‐MYC. Fig. S7. miR‐196a effects are mediated via c‐MYC. Fig. S8. MYC/TERT/NFκB axis is hyperactive in BE patients with high risk of developing EAC. Table S1. Primers used in this study. [file MOL2-19-3305-s001.zip › mol270048-sup-0001-DataS1.pdf]

# Figure S1

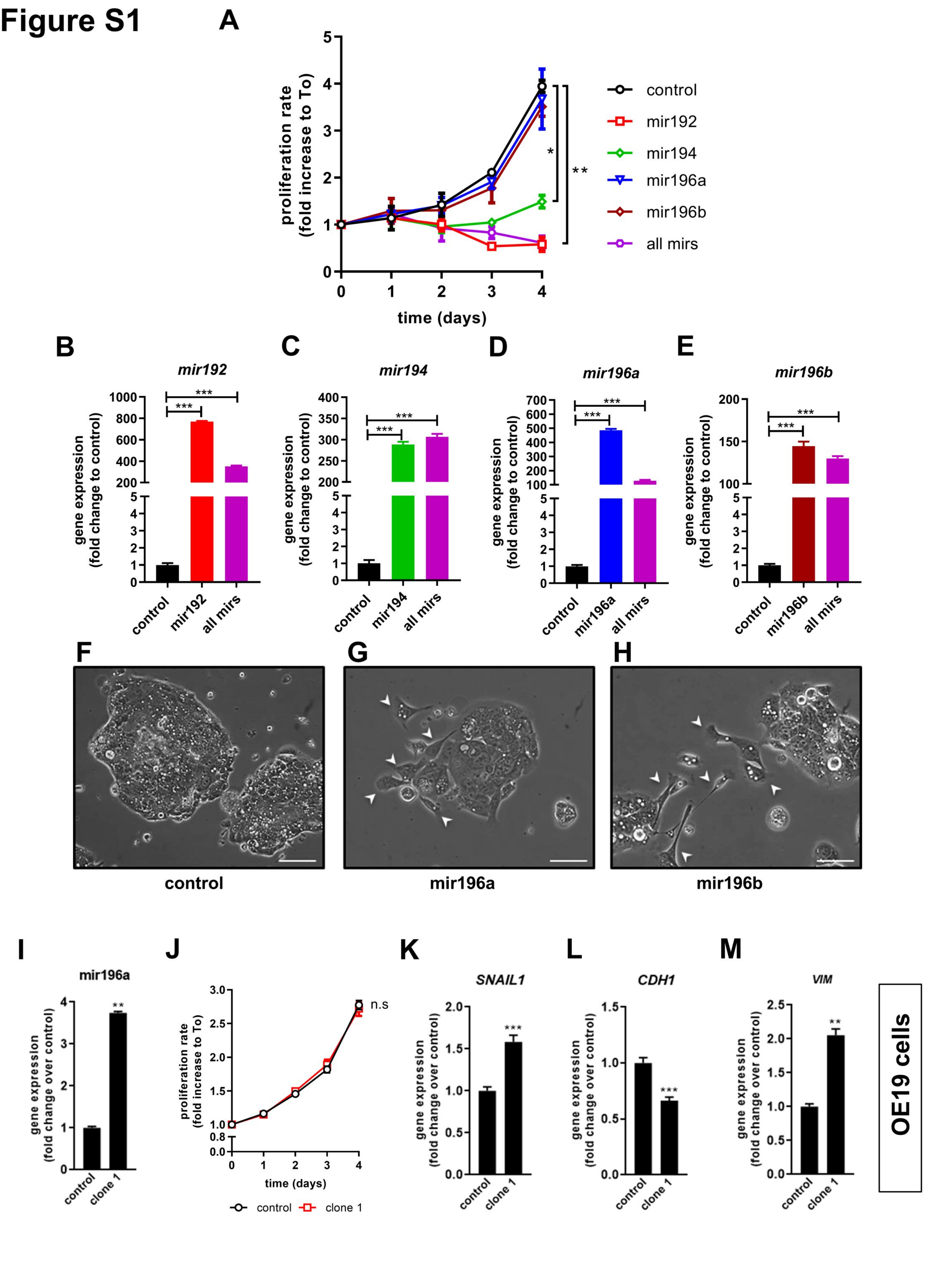
A
proliferation rate (fold increase to To)
time (days)
control
mir192
mir194
mir196a
mir196b
all mirs
*
**
B
mir192
C
mir194
D
mir196a
E
mir196b
gene expression (fold change to control)
***
control
all mirs
F
G
H
control
mir196a
mir196b
I
mir196a
gene expression (fold change over control)
**
clone 1
J
n.s
proliferation rate (fold increase to To)
time (days)
control
clone 1
K
SNAIL1
L
CDH1
M
VIM
OE19 cells

# Figure S2

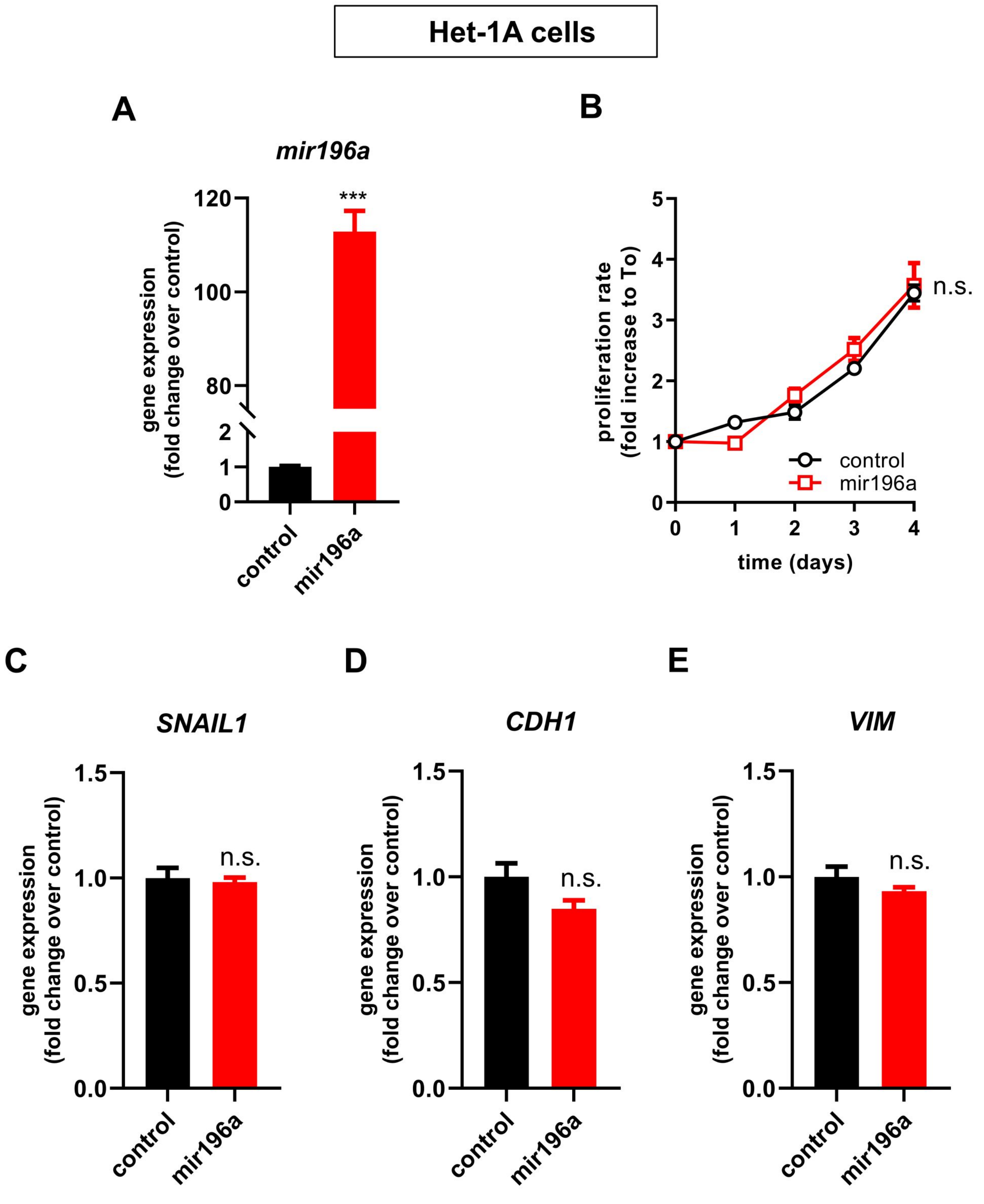

Het-1A cells
A
mir196a
***
gene expression
(fold change over control)
120
100
80
2
1
0
control
mir196a
B
proliferation rate
(fold increase to To)
5
4
3
2
1
0
n.s.
control
mir196a
0
1
2
3
4
time (days)
C
SNAIL1
D
CDH1
E
VIM
gene expression
(fold change over control)
1.5
1.0
0.5
0.0
n.s.
control
mir196a

# Figure S3

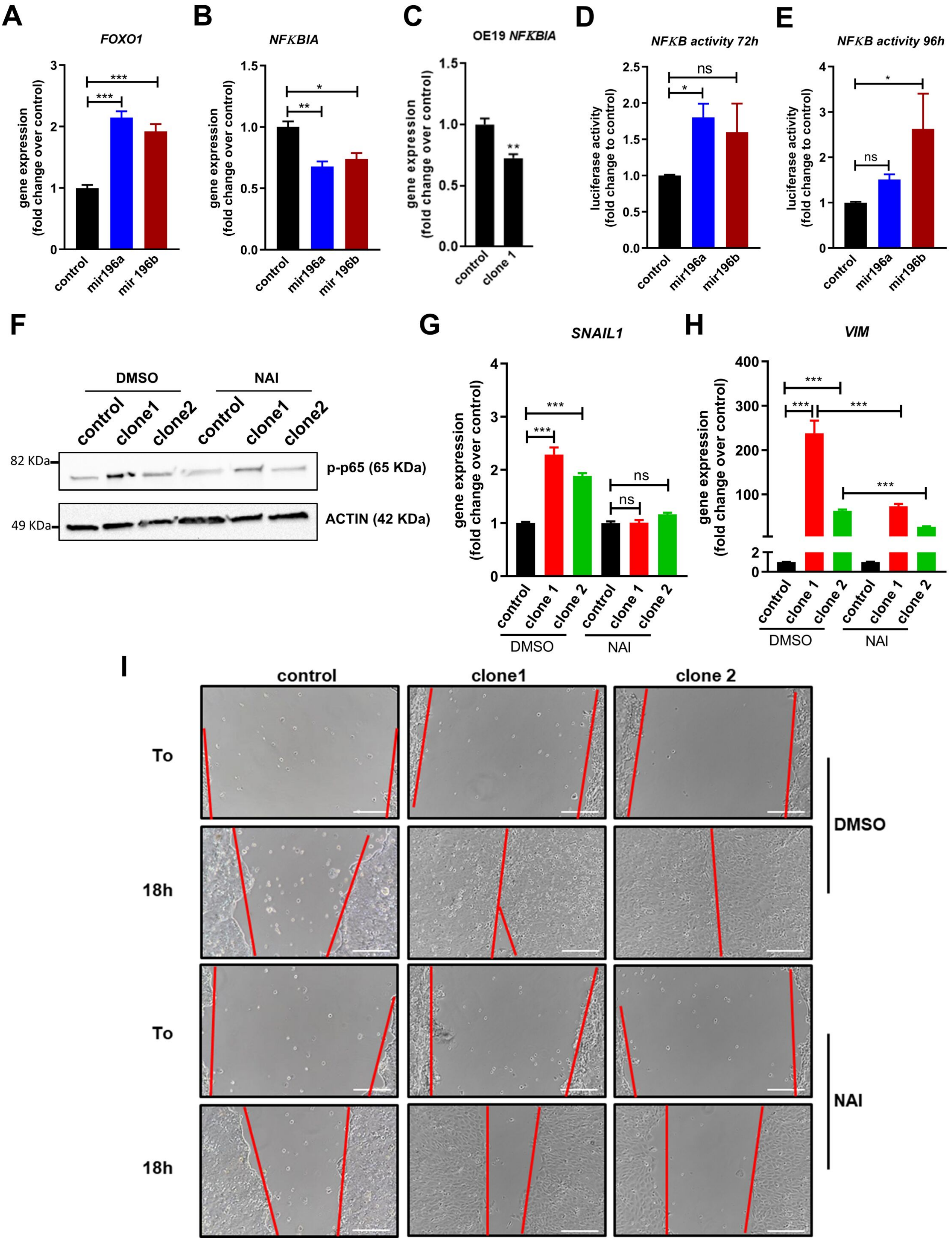
A
FOXO1
gene expression (fold change over control)
control
mir196a
mir 196b
***
***
B
NFKBIA
gene expression (fold change over control)
control
mir196a
mir 196b
**
*
C
OE19 NFKBIA
gene expression (fold change over control)
control
clone 1
**
D
NFKB activity 72h
luciferase activity (fold change to control)
control
mir196a
mir196b
*
ns
E
NFKB activity 96h
luciferase activity (fold change to control)
control
mir196a
mir196b
ns
*
F
DMSO
NAI
control
clone1
clone2
control
clone1
clone2
82 KDa
49 KDa
p-p65 (65 KDa)
ACTIN (42 KDa)
G
SNAIL1
gene expression (fold change over control)
control
clone 1
clone 2
control
clone 1
clone 2
DMSO
NAI
***
***
ns
ns
H
VIM
gene expression (fold change over control)
control
clone 1
clone 2
control
clone 1
clone 2
DMSO
NAI
***
***
***
***
I
control
clone1
clone 2
To
18h
To
18h
DMSO
NAI

# Figure S4

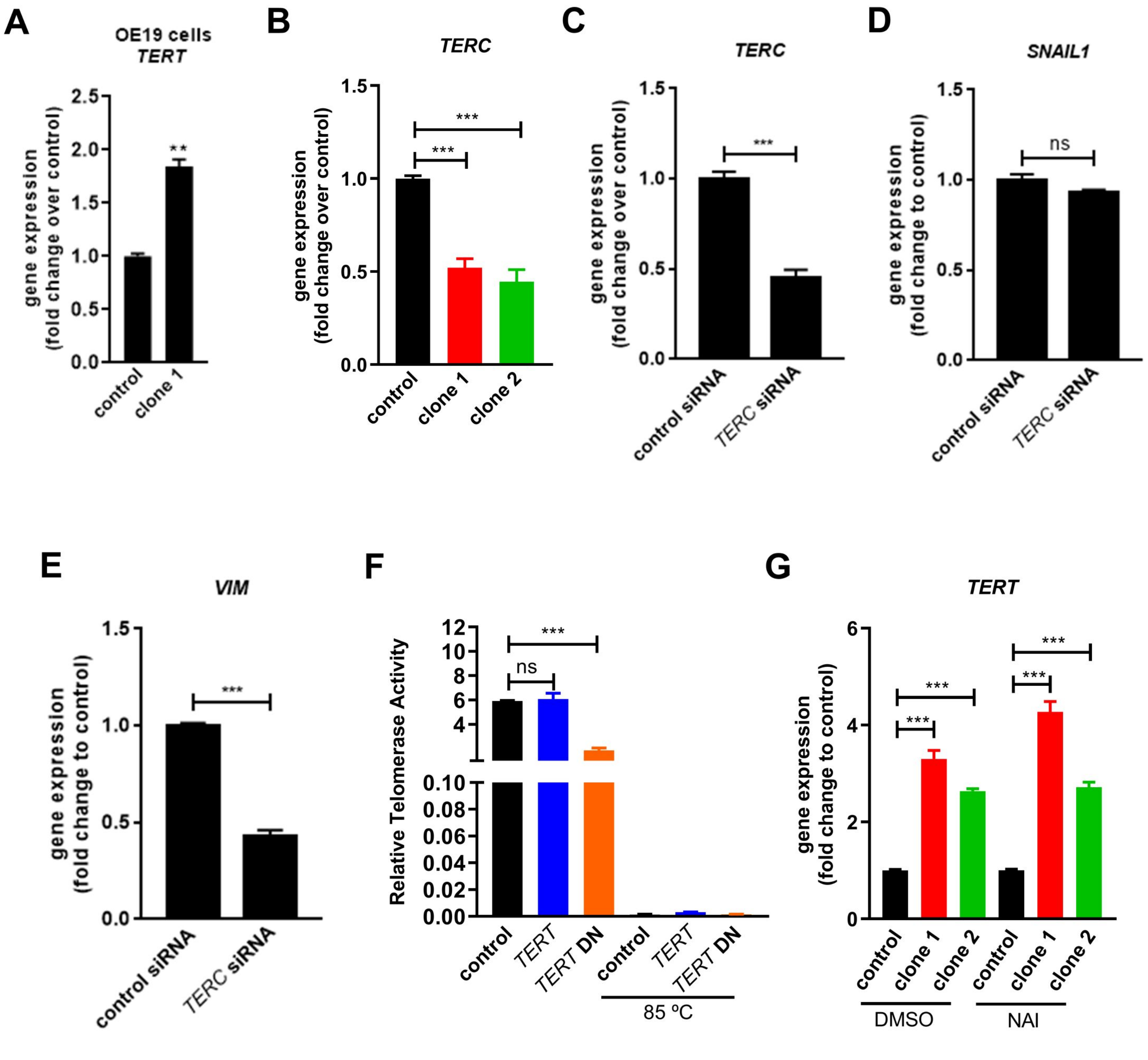
A
OE19 cells
TERT
gene expression
(fold change over control)
2.5
2.0
1.5
1.0
0.5
0.0
**
control
clone 1
B
TERC
gene expression
(fold change over control)
1.5
1.0
0.5
0.0
***
***
control
clone 1
clone 2
C
TERC
gene expression
(fold change over control)
1.5
1.0
0.5
0.0
***
control siRNA
TERC siRNA
D
SNAIL1
gene expression
(fold change to control)
1.5
1.0
0.5
0.0
ns
control siRNA
TERC siRNA
E
VIM
gene expression
(fold change to control)
1.5
1.0
0.5
0.0
***
control siRNA
TERC siRNA
F
Relative Telomerase Activity
12
10
8
6
4
0.10
0.08
0.06
0.04
0.02
0.00
***
ns
control
TERT
TERT DN
control
TERT
TERT DN
85 ºC
G
TERT
gene expression
(fold change to control)
6
4
2
0
***
***
***
***
control
clone 1
clone 2
control
clone 1
clone 2
DMSO
NAI

# Figure S5

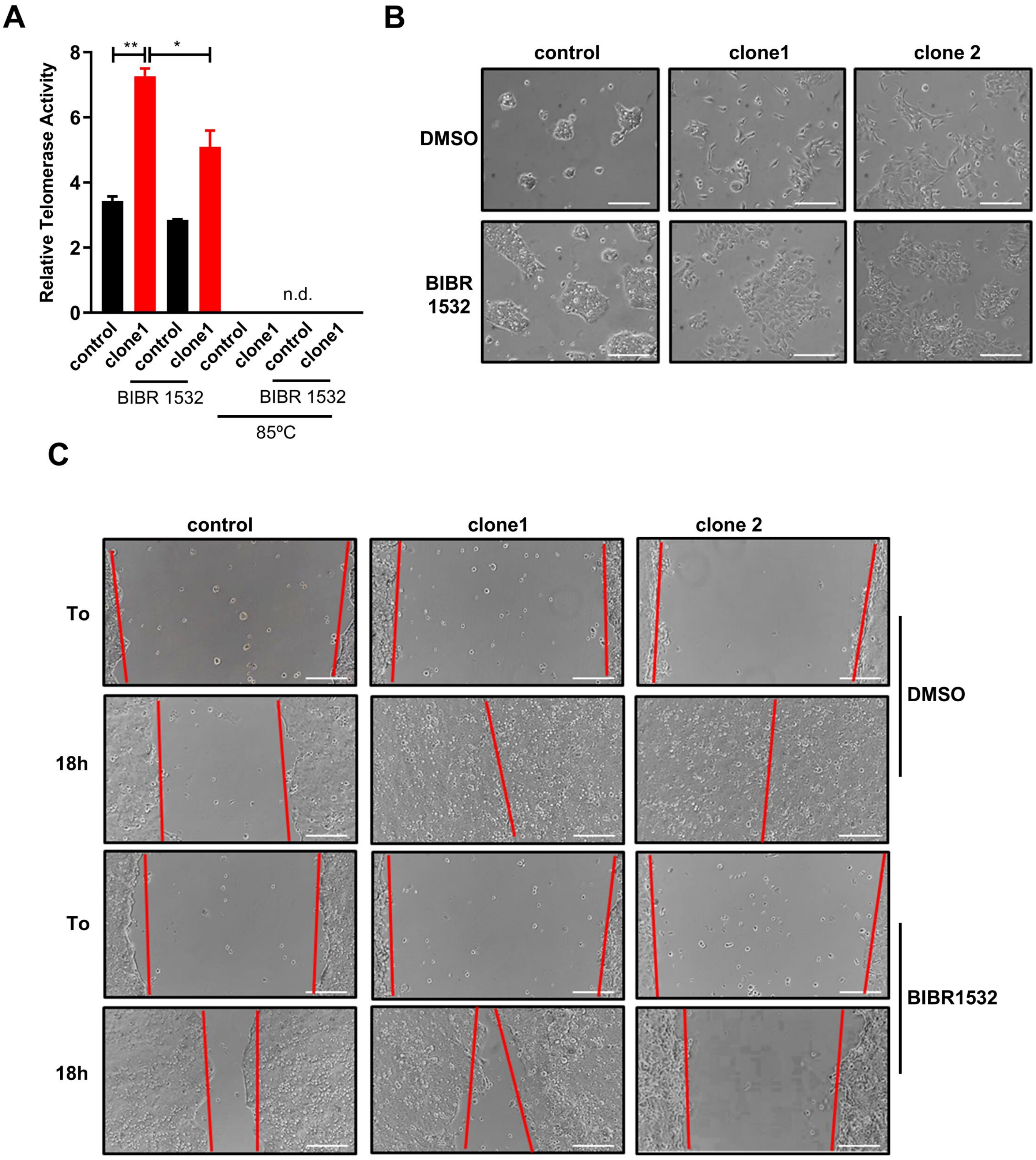
A
B
C
Relative Telomerase Activity
8
6
4
2
0
**
*
n.d.
control
clone1
control
clone1
control
clone1
control
clone1
BIBR 1532
BIBR 1532
85ºC
control
clone1
clone 2
DMSO
BIBR 1532
To
18h
To
18h
DMSO
BIBR1532

# Figure S6

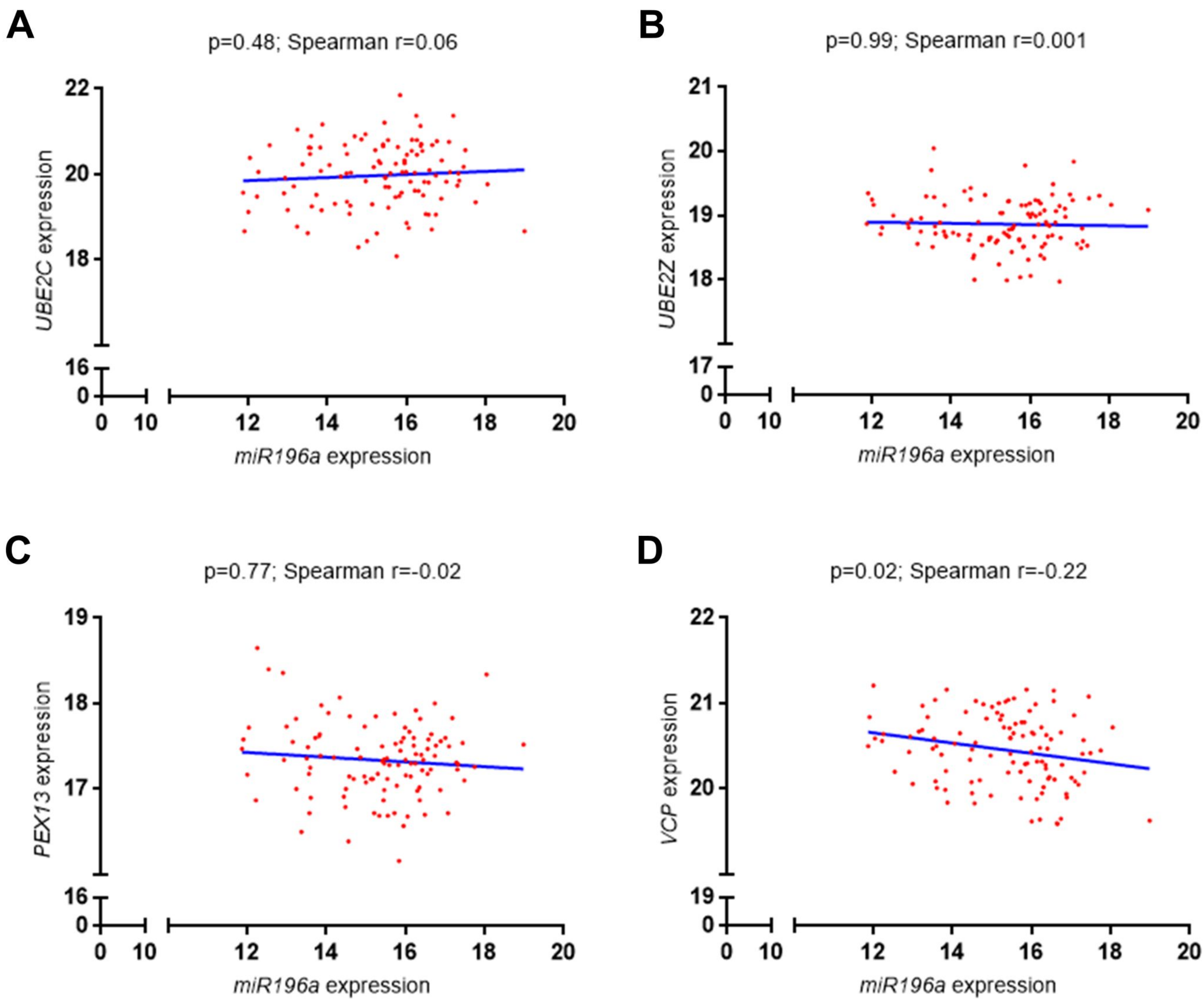
A
p=0.48; Spearman r=0.06
UBE2C expression
miR196a expression
B
p=0.99; Spearman r=0.001
UBE2Z expression
miR196a expression
C
p=0.77; Spearman r=-0.02
PEX13 expression
miR196a expression
D
p=0.02; Spearman r=-0.22
VCP expression
miR196a expression

# Figure S7

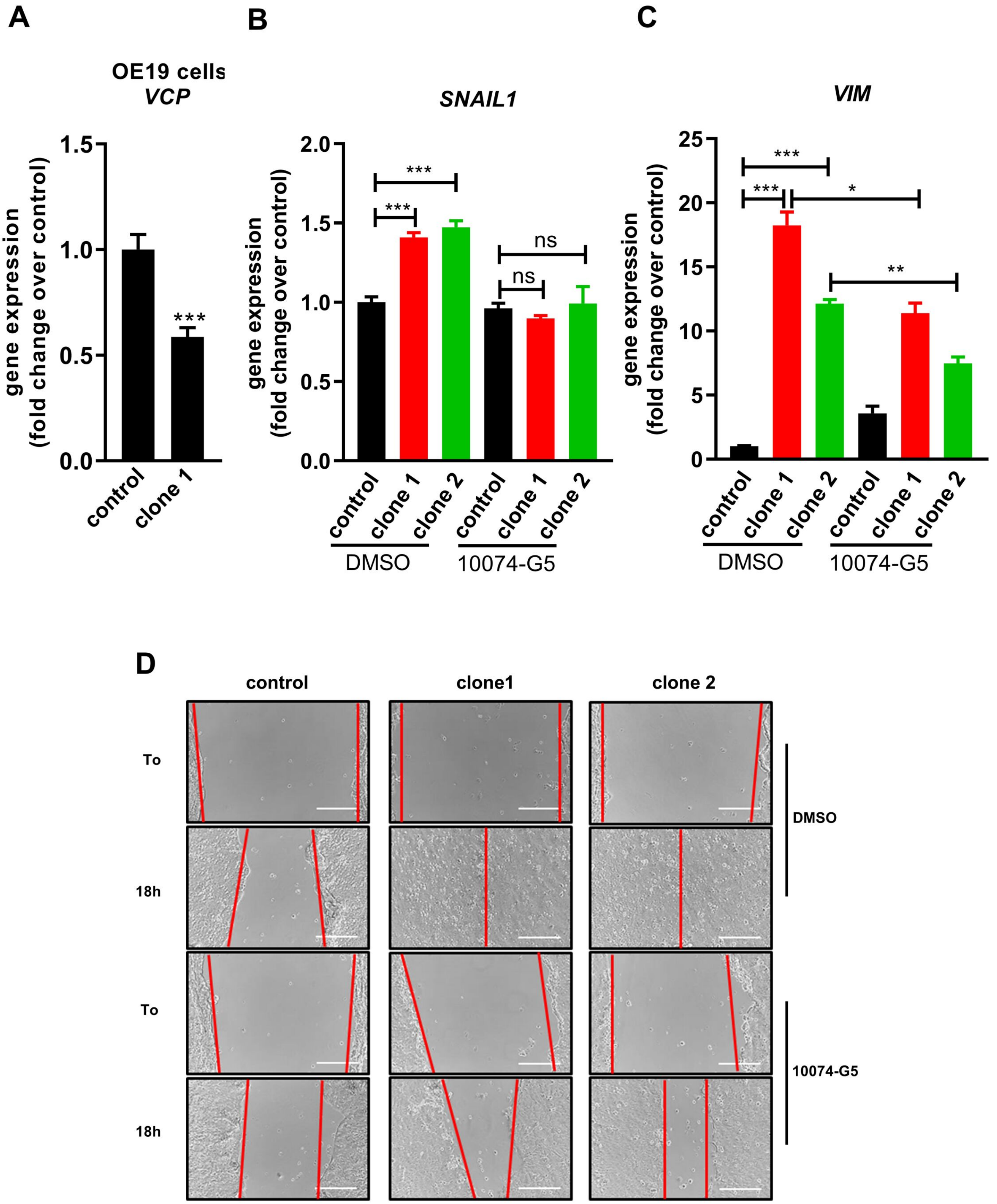
A
OE19 cells
VCP
gene expression
(fold change over control)
0.0
0.5
1.0
1.5
***
control
clone 1
B
SNAIL1
gene expression
(fold change over control)
0.0
0.5
1.0
1.5
2.0
***
***
ns
ns
control
clone 1
clone 2
control
clone 1
clone 2
DMSO
10074-G5
C
VIM
gene expression
(fold change over control)
0
5
10
15
20
25
***
***
*
**
control
clone 1
clone 2
control
clone 1
clone 2
DMSO
10074-G5
D
control
clone1
clone 2
To
18h
To
18h
DMSO
10074-G5

# Figure S8

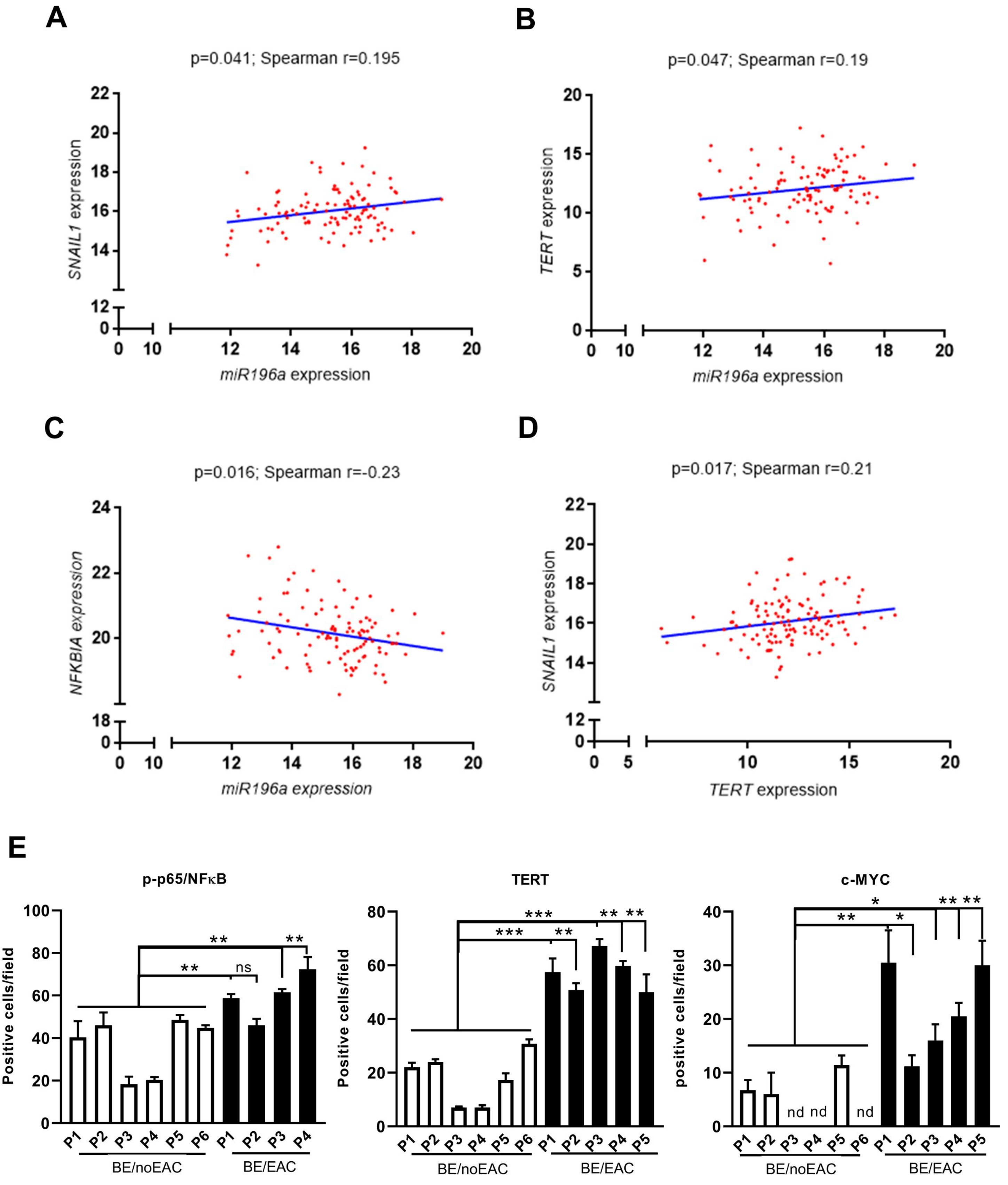

A
p=0.041; Spearman r=0.195
SNAIL1 expression
miR196a expression
B
p=0.047; Spearman r=0.19
TERT expression
miR196a expression
C
p=0.016; Spearman r=-0.23
NFKBIA expression
miR196a expression
D
p=0.017; Spearman r=0.21
SNAIL1 expression
TERT expression
E
p-p65/NFκB
Positive cells/field
BE/noEAC
BE/EAC
ns
TERT
Positive cells/field
BE/noEAC
BE/EAC
c-MYC
positive cells/field
nd
BE/noEAC
BE/EAC

**Supplemental Figure 1. microRNA (miRNA) 196 family induces a phenotype switch in OE33 cells and increased expression of epithelial to mesenchymal transition markers in OE19 cells.** (*A*) Proliferation rates of OE33 transiently transfected with the indicated miRNAs or all of them at the same time (all mirs). The statistical analysis showed corresponds to control cells (black line) *versus* mir-194a (green line), control cells *versus* mir-192 (red line) and control cells *versus* all mirs (purple). (*B-E*). Overexpression of the indicated miRNAs in these OE33 cells. (*F-H*) Images showing the phenotype switch in OE33 cells transiently transfected with miR-196a and miR-196b. Scale bar, 100µm. (*I-M*) Overexpression of miR196a in OE19 cells does not increase cell proliferation, increases *SNAIL1* and *VIMENTIN* (*VIM*) gene expression, and reduces *CDH1* mRNA levels. Data are mean + standard error of the mean of 3 independent experiments. * = $P< 0.05$, ** = $P < 0.01$ and *** = $P< 0.001$ for analysis of variance (ANOVA), plus Bonferroni post-test. n.s.: not significant.

**Supplemental Figure 2. Overexpression of miR-196a in non-transformed esophagus epithelial) Het-1A cells does not induce aggressiveness traits.** miR-196a overexpression in Het-1A cell line (*A*) does not increase cell proliferation (*B*) nor alters the mRNA levels of *SNAIL1*, *CDH1* and *VIMENTIN* (*VIM*) (*C-E*). Data are mean + standard error of the mean of 3 independent experiments. *** = $P< 0.001$ for Mann-Whitney test. n.s.: not significant.

**Supplemental Figure 3.miR-196a effects are mediated by NFκB signaling pathway.**

qPCRs showing the expression of *FOXO-1* in OE33 cells (*A*), *NFKBIA* (*B*) in OE33 cells transiently overexpressing miR-196a and miR-196b and in OE19 overexpressing cells (*C*). NFκB activity in cells transiently overexpressing miR-196a and miR-196b at 72h (*D*) and 96h (*E*) post-transfection. (*F*) Representative western blot of the phospho-

p65/RelA (p-p65) protein amounts in OE33 miR-196a overexpressing clones treated with 10 μM of NFκB inhibitor (NAI) or DMSO as control. (*I*) Images of the scratch-wound assays of miR-196a overexpressing clones and control cells after inhibition of NFκB for 18h. Red lines indicate the border of the cell layer. Scale bar, 200μm. Data are mean + standard error of the mean of 3 independent experiments. * = $P< 0.05$, ** = $P < 0.01$ and *** = $P< 0.001$ analysis of variance (ANOVA), plus Bonferroni post-test in *A*, *B D*, *E*, *G* and *H*, and for Mann-Whitney test in *C*. ns: not significant.

**Supplemental Figure 4. miR-196a effects are not mediated by *TERC*.** (*A*) OE19 overexpressing miR196a shows increased *TERT* gene expression. (*B*) Gene expression of *TERC* in OE33 clones overexpressing miR-196a. Gene expression of *TERC* (*C*), *SNAIL-1* (*D*) and *VIMENTIN* (*VIM*) (*E*) in OE33 parental cells transfected with siRNA targeting *TERC*. (*F*) Quantitative Telomere Repeats Amplification Protocol (QTRAP) assay for OE33 clones overexpressing either *TERT* or a dominant negative form of *TERT* (*TERT-DN*). (*G*) *TERT* expression in miR-196a overexpressing clones treated with 10 μM of NAI inhibitor or DMSO for 18h. Data are mean + standard error of the mean of 3 independent experiments. ** = $P < 0.01$ and *** = $P< 0.001$ analysis of variance (ANOVA), plus Bonferroni post-test in *B*, *F* and *G*, and for Mann-Whitney test in *A*, *C*, *D* and *E*. ns: not significant.

**Supplemental Figure 5. miR-196a effects are mediated by *TERT*.** (*A*) Quantitative Telomere Repeats Amplification Protocol (QTRAP) assay for OE33 clone1 treated with 20 μM of both temolerase activity and TERT non-canonical function inhibitor BIBR1532 or DMSO as control for 18h. (*B*) Images of the phenotype reversal of miR-196a overexpressing clones upon treatment with BIBR1532 or DMSO as control. Scale bar, 100μm. (*C*) Images of the scratch-wound assays of miR-196a overexpressing

clones and control cells after BIBR1532 inhibition. Red lines indicate the border of the cell layer. Scale bar, 200μm. Data are mean + standard error of the mean of 3 independent experiments. * = $P < 0.05$ and ** = $P < 0.01$ analysis of variance (ANOVA), plus Bonferroni post-test in *A*. n.d.; not detected.

**Supplemental Figure 6. miR-196a effects are mediated via c-MYC.** (*A-D*) Correlation between the expression of miR-196a and protein degradation-related miR-196a-predicted targets in a The Cancer Genome Atlas (TCGA) cohort of 109 patients of esophageal cancer.

**Supplemental Figure 7. miR-196a effects are mediated via c-MYC.** (*A-B*) Treatment of OE33 cells overexpressing miR-196a with 50 μM of c-MYC inhibitor (10074-G5) for 18h reduced the increment of both *SNAIL1* and *VIMENTIN* (*VIM*) gene expression. (*C*) Overexpression of miR196a in OE19 cells down-regulates the expression of *VCP*. (*D*) Images of the scratch-wound assays of miR-196a overexpressing clones and control cells after c-MYC inhibition for 18h. Red lines indicate the border of the cell layer. Scale bar, 200μm. Data are mean + standard error of the mean of 3 independent experiments. * = $P < 0.05$, ** = $P < 0.01$ and *** = $P < 0.001$ analysis of variance (ANOVA), plus Bonferroni post-test in *A* and *B*, and for Mann-Whitney test in *C*. n.s.; not significant.

**Supplemental Figure 8. MYC/TERT/NFκB axis is hyperactive in BE patients with high risk of developing EAC.** (*A-D*) Correlation between the expression of miR-196a and *SNAIL1*, miR-196a and *TERT*, miR-196a and *NFKBIA* and between *TERT* and *SNAIL1* in The Cancer Genome Atlas (TCGA) cohort of 109 patients of esophageal cancer. (*E*) Quantification of the immunochemistries for phospho-p65/RelA (p-p65NFκB), TERT and MYC of the different BE patients (showed by individual).

Statistical analysis was performed by analysis of variance (ANOVA), plus Bonferroni post-test comparing each BE/EAC patient to the average of all the BE/noEAC. * = $P<$ 0.05, ** = $P < 0.01$ and *** = $P< 0.001$. P1 to P6: patient 1 to patient 6. nd: non detected. ns: not significante

**Supplemental Table 1. Primers used in this study.**

| Name | Sequence (5´ to 3´) | Used for |
|---|---|---|
| Qiagen miR-192 Primer assay | n/a | miRNA expression |
| Qiagen miR-194 Primer assay | n/a | miRNA expression |
| Qiagen miR-196a Primer assay | n/a | miRNA expression |
| Qiagen miR-196b Primer assay | n/a | miRNA expression |
| Qiagen U6 Primer assay | n/a | miRNA expression |
| SNAIL Fq | CACAGGACTTTGATGAAGAC | gene expression |
| SNAIL Rq | CTCTGGATACAAAAACCCAC | gene expression |
| e-CAD Fq | TACATCTCCCTTCACAGC | gene expression |
| e-CAD Rq | ATAGATTCTTGGGTTGGGTC | gene expression |
| VIM Fq | GGAAACTAATCTGGATTCACTC | gene expression |
| VIM Rq | CATCTCTAGTTTCAACCGTC | gene expression |
| miR196a F | AATTGGGCCCACCCCCTTCCCTTCTCCTC | miR196a cloning |
| miR196a R | AATTGGGCCCACAGCTTGTCCTCCTTGGTC | miR196a cloning |
| FOXO1 Fq | CCAGCCAAACTACCAAAAATA | gene expression |
| FOXO1 Rq | GAGGAGAGTCAGAAGTCAGCAAC | gene expression |
| IKBa Fq | CGGGTCCTGCACTTGGCCATC | gene expression |
| IKBa Rq | GTCCGGCCATTACAGGGCTC | gene expression |
| TERT Fq | AGAACGCAGGGATGTC | gene expression |
| TERT Rq | CAGCTTGCGCAGGAATG | gene expression |
| TERC Fq | CCCTAACTGAGAAGGGCGTA | gene expression |
| TERC Rq | GCTCTAGAATGAACGGTGGAA | gene expression |
| MYC Fq | TGAGGAGGAACAAGAAGATG | gene expression |
| MYC Rq | ATCCAGACTCTGACCTTTTG | gene expression |
| VCP Fq | TAGAGGAATCCTGCTTTACG | gene expression |
| VCP Rq | CCATTGATCAAGAAGAAGAAGG | gene expression |
| VCP3UTRF | AAGGTCTAGAGCCTGCCTGGACCTTGTTC | VCP3´UTR cloning |
| VCP3UTRR | AAGGTCTAGAAGGTGGAGGGATGCCATATT | VCP3´UTR cloning |
| VCP3UTRmutF | TTTATATAGAGAGTATAATCACAAGCAGTT | VCP3´UTR miR-196a binding deletion |
| VCP3UTRmutR | AACTGCTTGTGATTATACTCTCTATATAAA | VCP3´UTR miR-196a binding deletion |
| GAPDH Fq | CTTTTGCGTCGCCAG | gene expression |
| GAPDH Rq | TTGATGGCAACAATATCCAC | gene expression |
