# Supplementary material for: MicroRNA 196a contributes to the aggressiveness of esophageal adenocarcinoma through the MYC/TERT/NFκB axis
Source: Mol Oncol. 2025 Sep 16;19(11):3305–24. doi: 10.1002/1878-0261.70048 (PMC12591326; doi:10.1002/1878-0261.70048)
Supplement: Supplementary file 1 — Fig. S1. microRNA (miRNA) 196 family induces a phenotype switch in OE33 cells and increased expression of epithelial‐to‐mesenchymal transition markers in OE19 cells. Fig. S2. Overexpression of miR‐196a in non‐transformed esophagus epithelial Het‐1A cells does not induce aggressiveness traits. Fig. S3. miR‐196a effects are mediated by NFκB signaling pathway. Fig. S4. miR‐196a effects are not mediated by TERC. Fig. S5. miR‐196a effects are mediated by TERT. Fig. S6. miR‐196a effects are mediated via c‐MYC. Fig. S7. miR‐196a effects are mediated via c‐MYC. Fig. S8. MYC/TERT/NFκB axis is hyperactive in BE patients with high risk of developing EAC. Table S1. Primers used in this study. [file MOL2-19-3305-s001.zip › mol270048-sup-0002-TableS1.docx]

**Supplemental Table 1. Primers used in this study.**

| **Name** | **Sequence (5´ to 3´)** | **Used for** |
| --- | --- | --- |
| Qiagen miR-192 Primer assay | n/a | miRNA expression |
| Qiagen miR-194 Primer assay | n/a | miRNA expression |
| Qiagen miR-196a Primer assay | n/a | miRNA expression |
| Qiagen miR-196b Primer assay | n/a | miRNA expression |
| Qiagen U6 Primer assay | n/a | miRNA expression |
| SNAIL Fq | CACAGGACTTTGATGAAGAC | gene expression |
| SNAIL Rq | CTCTGGATACAAAAACCCAC | gene expression |
| e-CAD Fq | TACATCTCCCTTCACAGC | gene expression |
| e-CAD Rq | ATAGATTCTTGGGTTGGGTC | gene expression |
| VIM Fq | GGAAACTAATCTGGATTCACTC | gene expression |
| VIM Rq | CATCTCTAGTTTCAACCGTC | gene expression |
| miR196a F | AATTGGGCCCACCCCCTTCCCTTCTCCTC | miR196a cloning |
| miR196a R | AATTGGGCCCACAGCTTGTCCTCCTTGGTC | miR196a cloning |
| FOXO1 Fq | CCAGCCAAACTACCAAAAATA | gene expression |
| FOXO1 Rq | GAGGAGAGTCAGAAGTCAGCAAC | gene expression |
| IKBa Fq | CGGGTCCTGCACTTGGCCATC | gene expression |
| IKBa Rq | GTCCGGCCATTACAGGGCTC | gene expression |
| TERT Fq | AGAACGCAGGGATGTC | gene expression |
| TERT Rq | CAGCTTGCGCAGGAATG | gene expression |
| TERC Fq | CCCTAACTGAGAAGGGCGTA | gene expression |
| TERC Rq | GCTCTAGAATGAACGGTGGAA | gene expression |
| MYC Fq | TGAGGAGGAACAAGAAGATG | gene expression |
| MYC Rq | ATCCAGACTCTGACCTTTTG | gene expression |
| VCP Fq | TAGAGGAATCCTGCTTTACG | gene expression |
| VCP Rq | CCATTGATCAAGAAGAAGAAGG | gene expression |
| VCP3UTRF | AAGGTCTAGAGCCTGCCTGGACCTTGTTC | VCP3´UTR cloning |
| VCP3UTRR | AAGGTCTAGAAGGTGGAGGGATGCCATATT | VCP3´UTR cloning |
| VCP3UTRmutF | TTTATATAGAGAGTATAATCACAAGCAGTT | VCP3´UTR miR-196a binding deletion |
| VCP3UTRmutR | AACTGCTTGTGATTATACTCTCTATATAAA | VCP3´UTR miR-196a binding deletion |
| GAPDH Fq | CTTTTGCGTCGCCAG | gene expression |
| GAPDH Rq | TTGATGGCAACAATATCCAC | gene expression |
